# A scoping review of social media in child, adolescents and young adults: research findings in depression, anxiety and other clinical challenges

**DOI:** 10.1192/bjo.2023.523

**Published:** 2023-08-11

**Authors:** Donald M. Hilty, Dorothy Stubbe, Alastair J. McKean, Pamela E. Hoffman, Isheeta Zalpuri, Myo T. Myint, Shashank V. Joshi, Murat Pakyurek, Su-Ting T. Li

**Affiliations:** Department of Psychiatry and Behavioral Sciences, University of California, Davis, California, USA; and Mental Health, Veterans Affairs Northern California Health Care System, California, USA; Child Study Center, Yale School of Medicine, Connecticut, USA; Department of Psychiatry and Psychology, Mayo Clinic, Minnesota, USA; Department of Psychiatry & Behavioral Science, Yale School of Medicine, Connecticut, USA; Department of Psychiatry & Behavioral Science, Stanford University Medical Center, California, USA; Department of Psychiatry & Behavioral Science, Tulane University School of Medicine, Louisiana, USA; Division of Child and Adolescent Psychiatry, University of California, Davis School of Medicine, California, USA; Department of Pediatrics, University of California, Davis School of Medicine, California, USA

**Keywords:** Social media, adolescents, children, suicide, youth

## Abstract

**Background:**

Social media and other technologies are reshaping communication and health.

**Aims:**

This review addresses the relationship between social media use, behavioural health conditions and psychological well-being for youth aged <25 years.

**Method:**

A scoping review of 11 literature databases from 2000 to 2020 explored research studies in youth in five areas: clinical depression and anxiety, quantitative use, social media mode, engagement and qualitative dimensions and health and well-being.

**Results:**

Out of 2820 potential literature references, 140 met the inclusion criteria. The foci were clinical depression and anxiety disorders (*n* = 78), clinical challenges (e.g. suicidal ideation, cyberbullying) (*n* = 34) and psychological well-being (*n* = 28). Most studies focused on Facebook, Twitter, Instagram and YouTube. Few studies are longitudinal in design (*n* = 26), had comparison groups (*n* = 27), were randomised controlled trials (*n* = 3) or used structured assessments (*n* = 4). Few focused on different youth and sociodemographic populations, particularly for low-income, equity-seeking and deserving populations. Studies examined association (*n* = 120; 85.7%), mediating (*n* = 16; 11.4%) and causal (*n* = 4; 2.9%) relationships. Prospective, longitudinal studies of depression and anxiety appear to indicate that shorter use (≤3 h/day) and purposeful engagement is associated with better mood and psychological well-being. Depression may predict social media use and reduce perception of support. Findings provide families, teachers and providers ways to engage youth.

**Conclusions:**

Research opportunities include clinical outcomes from functional perspective on a health continuum, diverse youth and sociodemographic populations, methodology, intervention and privacy issues. More longitudinal studies, comparison designs and effectiveness approaches are also needed. Health systems face clinical, training and professional development challenges.

Children, adolescents and young adults under 25 years of age (i.e. youth) are raised in an increasingly digitalised society, with technology as an integral part of daily life; some researchers suggest 30 years of age as a limit of youth, but there is not consensus on this.^1^ Social media is very attractive to youth as it is portable and offers ever-changing, immersive, diverse, individualised social engagement. The following social media platforms have launched since 2000: networks Facebook (2004), Twitter (2006) and LinkedIn (2002); media-sharing networks Instagram (2010), Snapchat (2011) and YouTube (2005); discussion forums Reddit (2005), Quora (2009) and Digg (2004); and bookmarking and content curation networks Pinterest (2010) and Flipboard (2010). Youth mostly use YouTube (81%) and Facebook (69%).^1^ Instagram and Snapchat are also commonly used, with the latter as the most important social network for 44% of youth.

Youth are vulnerable in many ways, and may need supervision with social media because of their limited ability to self-regulate, vulnerability to peer pressure and susceptibility to sharing personal information.^2^ Teenagers acknowledge social media's role in helping build their social connections and expose them to a diverse world, and cite concerns around the social pressure that it generates.^3^ Most (65%) parents worry about their children spending too much time in front of screens, and its impact on mental and physical health, safety, well-being, social development and family dynamics.^4^ The USA Children's Online Privacy Protection Act has effectively guided participants since 1998, if and when those aged ≤13 years adhere to parental/guardian permission.^5^

## Current state

This review attempts to describe and consider improvements to the literature about social media use in youth and young adults, as there are many things that are still unknown despite past studies and reviews.^3–16^ How social media is used may make a difference in how it is experienced – from browsing through content to posting content to directed communication (e.g. conversational or liking content) – and if this is self-reported, methods are needed to monitor and verify. The positive and negative effects of social media related to clinical populations (i.e. normal versus problematic use) are not well described. Past studies and reviews are limited by the lack of consensus on definitions of terminology (e.g. normal versus problematic use, sexting, cyberbullying);^3,4,6^ the quality of social-media-specific assessment tools and the rigor of other tools applied to social media; quality of study designs (e.g. cross-sectional or short-term designs that limit evaluation of outcomes) and summarising data, with emphasis on the better designs. Prior reviews found that social media use is negatively correlated with well-being,^7–12^ but the linkage to depression and/or lower self-esteem is not clear.^11–15^ Many reviews reported both negative effects (low mood or esteem, decreased offline prosocial activity, overuse, impulsivity) and positive effects (developing friends, feeling connected, social capital).^16^ Unfortunately, many prior reviews did not clarify the relationship between social media and behavioural health issues (i.e. associative, mediating versus causal relationships).^8,12^ Ideally, more data from across the world is needed, rather than studies from a few countries.

This scoping review explores the question ‘What is the nature of the relationship (i.e. association, mediation, causation and/or other) between social media use in children/adolescents/young adults, psychopathology and mental and/or behavioural health conditions or problems?’. This review is intended to assist providers in educating adolescent/young adult patients and their families in how to best interact with social media. The review has several aims.

- To summarise findings of the relationship (association, mediation, causation) between social media use in children/adolescents/young adults, psychopathology and mental and/or behavioural health conditions or problems.
- To explore the unique challenges, effects and benefits of social media use by youth, related to clinical populations for depression and anxiety (Supplementary Table 1 available at https://doi.org/10.1192/bjo.2023.523);^17–90^ clinical challenges like cyberbullying, sexting and suicide (Supplementary Table 2);^91–123^ and health behaviour and well-being (Supplementary Table 3).^12,124–149^
- Based on the literature, to provide an approach for future clinical research and approaches for providers and health systems to social media in youth (Table 1).

**Table 1:** Approach for providers to social media use by youth and young adults: clinical questions and protective factors

Areas/questions	Prompts or specifics	Follow-up questions	Protective factors
Normalise, when possible, the use of electronics and devices Which do you use? How much time per day?	Teenagers spend an average of 6–8 h daily Options • Mobile phone • Computer/tablet • Gaming systems (X-Box, Wii, PlayStation) • Television • Other	Which do you use the most? Which is your favourite? Compared with the average teen, about how much screen time do you have daily? Do you use privacy settings? Have you lied about your age to gain access?	Purposeful networking/ communication Use focused on new friendships or building current relationship Emotionally stable, restrained or high harm avoidance Approachable, high social skills Serene, satisfied with life and high self-esteem
Screen for social networking accounts/profile. Which is your favourite? Why? Are you ‘friends’ with your parents or siblings? Do your parents limit your time? Daytime Evening/night Do your parents have your passwords?	Facebook Twitter Instagram YouTube Tumblr Google Plus Pinterest Email Snapchat Video-sharing platforms Chat rooms MySpace Other	Do you use media to deal with stress? How often? Number of hours per day? Does media use cause you stress, and if so, how? What do you post? Photos, posts, phone, school, city, birthdate? Videos: what kind? Have you posted or received inappropriate photos? Have your posts caused problems for you, a friend or family member? How many friends do you have? Do you personally know all of the friends on your social media? Have you interacted with strangers online, and if so, do you use your real name? Have you had bad experiences?	Open discussion of use and high level of supervision Those who may need more supervision Low levels of psychopathology Anxiety Depression Not psychotic Not autism spectrum disorder Not attention-deficit hyperactivity disorder Males Younger Lower-income family (i.e. may need more structure and support)
Screen for positive aspects of internet and media use	Which specific sites have been helpful to you?	Have you gotten health or mental health information online? Have you used self-help sites? Have you made friends or gotten closer to a friend online? Have you used apps for wellness? Have you ever helped a friend who felt suicidal online or via text/chat?	Parallel positive family involvement/function Interested/involved Higher functioning and education Positive relationship role modelling Positive peer relations Opposed to bullying Supportive Friendly actions/ pranks
Screen for problematic use or risky behaviour	Online use disrupts your sleep Visiting sites with sensitive topics that you do not want others to know Meeting others online that you do not know Times you were involved with or seen bullying or harassment Sexting	How often do you stay up at night on media? Have you visited sites regarding: Weapons Porn Suicide Anorexia Other? Have you interacted with strangers online, and if so, do you use your real name and identification info? Have you planned to meet up with someone you met on the internet? Have you ever been bullied or harassed online? Or have you bullied or harassed others online? Have you regretted posts or do not want friends/parents to see your posts? Have you ever sexted?	Interest shown and support offered Structured periods of access Reward systems in place Regular parenting schedules Consistent availability for chatting and encouragement Lower stress High stimulation/activity Autonomy supported if earned

## Method

### Approach

The literature search was conducted from January 2000 to December 2020. The philosophical approach to the search was done according to the original six-stage process^150^ and updated modifications^151^ (purposeful research question, identifying relevant studies, selecting studies based on an iterative process, charting the data, analysis of findings and consultation from stakeholders). The Preferred Reporting Extension for Systematic Reviews and Meta-Analyses (PRISMA) for scoping reviews^152^ has additional suggestions for sources of information, the search and appraising data.

### Research question

This review addresses the overarching question: ‘What is the nature of the relationship between social media use, psychopathology and mental and/or behavioural health conditions or problems?’ The population of interest is children, adolescents and young adults (aged ≤25 years). Secondary questions are as follows.

- What social media is commonly used, in what ways and for what purpose(s) (i.e. approach, interest, motivation)?
- In what ways is social media helpful, neutral or negative related to clinical populations for depression and anxiety, and specific problems like cyberbullying, sexting and suicide?
- What is the relationship (i.e. association, mediation, causation and/or other) between social media (e.g. Facebook, Twitter, Instagram) and behavioural health?
- What methods of assessment, triage and approaches, interventions and professional development can help providers, parents, teachers and others in the community to help?

### Identifying relevant studies

Eleven databases were queried: PubMed/Medline, APA PsycNET, Cochrane Database of Systematic Reviews, EMBASE, PsycINFO, Web of Science and Scopus, Social Sciences Citation Index (SSCI), Centre for Reviews and Dissemination, Cochrane Central Register of Controlled Trials, Cumulative Index to Nursing and Allied Health Literature (CINAHL) and Google Scholar.

**Fig. 1:**
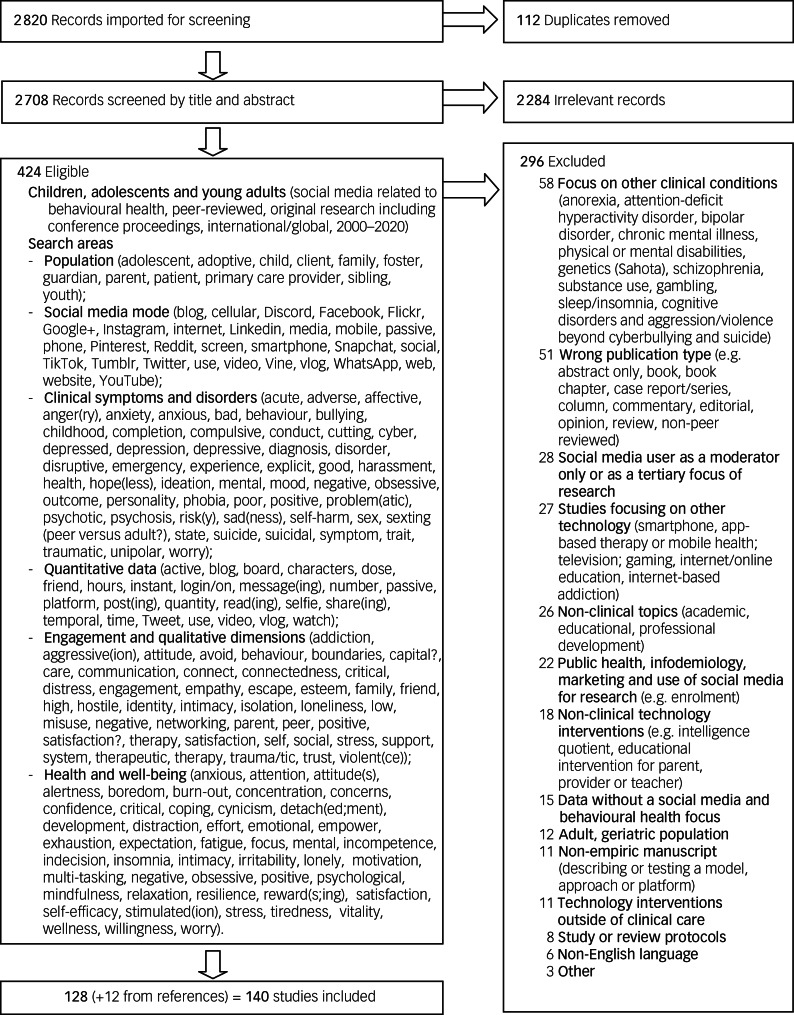
Search flow diagram for child, adolescent and young adult social media articles reviewed.

The search focused on youth (adolescent, child, children, high, junior, juvenile, middle, minor, secondary, teenager, youth) and social media use in five concept areas (Fig. 1): clinical depression and anxiety and problematic challenges (e.g. suicidal ideation, cyberbullying); quantitative data; social media mode; engagement and qualitative dimensions; and health and psychological well-being. Definitions were used based on consensus literature: bullying is a subset of aggressive behaviour that involves repeated and intentional attempts to damage/distress a weaker victim by a more powerful perpetrator;^153^ and sexting is sending or receiving of sexually explicit pictures, videos, or text messages via smartphone, digital camera or computer.^96^ Exclusion criteria included studies focusing on anorexia, attention-deficit hyperactivity disorder, physical or intellectual disabilities, genetics, substance use, gambling, sleep/insomnia, cognitive disorders and aggression/violence beyond cyberbullying and suicide) (Fig. 1).

### Study selection

One author (D.M.H.) screened titles and abstracts of potential references, excluding duplicates and those that did not meet the search criteria. Two authors (D.M.H., D.S.) reviewed the full text of remaining abstracts to find those meeting inclusion criteria; additional studies that met inclusion criteria were added from references.

### Data charting

A data-charting form was used to extract data, and notes were organised with a descriptive analytical method. The reviewers (D.M.H., D.S.) compared and consolidated information by using a modified content analysis with thematic components;^154^ a third author (A.J.M.) moderated any disagreement and a fourth author (S.-T.T.L.) analysed consistency of the approach. The information was shared with selected experts, their input summarised and themes extracted.

### Analysis, reporting and the meaning of findings

**Fig. 2:**
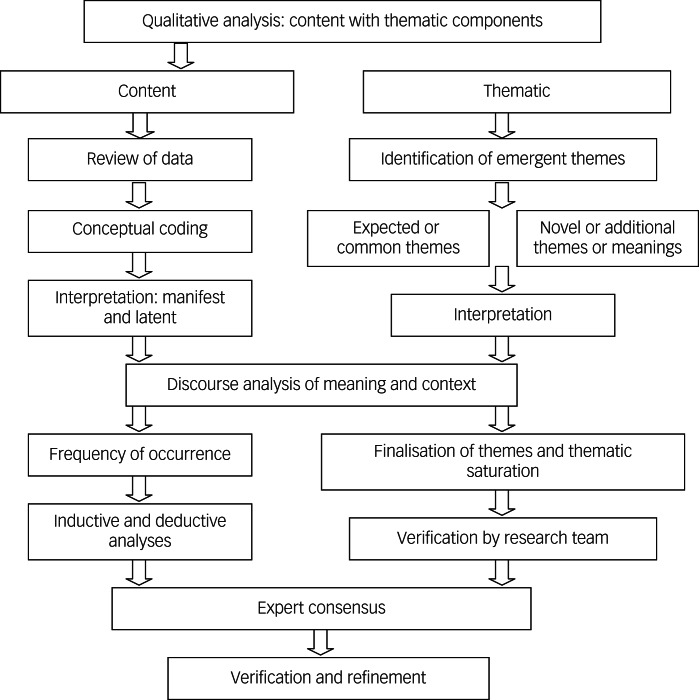
Qualitative steps to analyse disparate study populations, methodology and data.

Results were organised into tables, with key concepts and components outlined and described, partially based on excerpts from published topics. The studies varied considerably, and therefore were challenging to compare. Qualitative steps to analyse disparate populations, methods and data of studies were used (Fig. 2).^154^ Content, discourse and framework qualitative analysis techniques were to analyse findings from papers and classify, summarise and tabulate the behavioural data; discourse and thematic analyses were used to search for themes and patterns; and framework analysis was used to sift through, chart and sort data in accordance with key issues and themes a series of steps (e.g. indexing, charting, mapping and interpretation).^154^ Data in Supplementary Tables 1–3 are organised by study, sample size, population (e.g. country), objective and design, methods and measures, outcomes and clinical implications/challenges and training/research foci.

### Expert opinions and feedback

Expert opinions were solicited to review preliminary findings and suggest additional steps for improvement. A list of relevant experts was compiled from (a) behavioural health organisations across professions internationally; (b) technology-related special interest groups of organisations (e.g. American Telemedicine; Medical, Nursing and Informatics Associations); (c) educational and professional development organisations (e.g. Accreditation Council of Graduate Medical Education, American Academy of Child and Adolescent Psychiatry, American Academy of Pediatrics); (d) academic institutions and (e) researchers, authors, editors and editorial board members of journals related to social media.

Experts were invited by email (*N* = 24) and attended a live expert feedback session for discussion and feedback; completed a qualitative and quantitative five-item Likert scale survey (*n* = 20; 83.3%) and/or provided qualitative feedback via email (*n* = 4; 16.7%). The data charting and the search criteria plan were reviewed; their input did not suggest a search with additional terminology or otherwise change the scope. Input was summarised and themes were extracted to guide the organisation (e.g. headings in rows) and content (e.g. in the columns) of Table 1 and Supplementary Tables 1–3, based on previous work using consensus and modified Delphi processes.^154^ Results showed that the majority agreed or strongly agreed that the search strategy was effective using the research question (*n* = 21; 87.5%); it was systematic/thorough (20; 83.3%); and adequately scientific in methodology (*n* = 18; 75%); and ‘[The tables] are organised in a practical way to summarise social media study findings for providers, teachers and systems’ (*n* = 20; 83.3%), once more specific outcomes were entered in the final column of each.

## Results

### Literature overview

Out of 2820 potential literature references, 112 duplicates and 2284 studies that were outside of the scope of this review were excluded (Fig. 1). Full-text review of 424 articles revealed that 128 met full inclusion criteria; 12 additional studies were found within those, for a total of 140 studies.^12,17–95,97–149,155^ The studies focused on clinical populations for depression and anxiety,^71^ clinical challenges (e.g. suicidal ideation, cyberbullying)^27^ or psychological well-being.^21^ Studies were of children aged 12 years and younger (*n* = 1; 0.01%), adolescents (13–18 years) (*n* = 72; 54.1%) and young adults (19–25 years) (*n* = 48; 34.2%); the rest were aggregates of the above (*n* = 18; 12.9%). The overall mean age was 18.78 years. The most common social media studied were Facebook (*n* = 62), Twitter (*n* = 20), Instagram (*n* = 11), YouTube (*n* = 6) and MySpace (*n* = 5). Studies varied in identifying gender identity (*n* = 63; 45%), ethnicity and race (*n* = 42; 30%) or neither (*n* = 35; 25%).

Most studies were cross-sectional cohort studies using self-report questionnaires. Few studies were longitudinal in design,^19^ had comparison groups^20^ or were randomised controlled trials.^3^ Few studies used clinician/provider-administered instruments^2,20,30^ or structured assessments.^4,17,51,82,131^ Timing or temporal dimensions are generally quite limited and studies span across acute disorders, subacute symptoms and trait/personality factors among a wide variety of ethnic, clinical and non-clinical populations. Broadly speaking, the studies looked at associations (*n* = 120; 85.7%),^17–23,25–29,31–33,36–41,43–53,55–61,63–73,75,76,78–84,86–95,97–100,102,103,105,112,114–127,129–131,133–142,144,146,148,149,154,155^ and mediating (*n* = 16; 11.4%)^24,30,34,35,62,74,77,85,101,104,113,128,132,143,145^ and causal (*n* = 4; 2.9%)^42,54,104,147^ relationships between social media and behavioural health issues.

### Clinical populations, depression and anxiety

There were 78 studies of social media with outcomes in clinical populations and disorders (Supplementary Table 1). The mean age was 18.4 years (median 18 years) and included adolescents^42^ and young adults.^21^ The study populations were diverse in terms of ethnicity, but were predominately White, and 46 studies were ≥50% female. The mean sample size was 8332.4 (median 310). The most common social media sites studied were Facebook (*n* = 37), Twitter (*n* = 10), Instagram (*n* = 5), MySpace (*n* = 2) and YouTube (*n* = 2); two were on screen time.^13,33^

Cross-sectional and longitudinal studies^12,19,22,23,25,27,28,31,35,41,42,47,48,51,65,67,76,79,87,89^ of social media use and depression found that shorter periods of social media use (<3 h), particularly with purposeful or active engagement, are associated with better mood and psychological well-being, whereas longer periods of social media use predict depression (and often anxiety) or poorer psychological function^22,25,27,28,31,35,48,59,65,87^ (particularly browsing^30,89^), partly because of sleep disruptions.^47^ Cross-sectional and longitudinal studies are consistent with one prospective study that suggests a threshold effect around 3 h that has negative impact for many, but not all, users: low use-stable (80% at 3–4 h/day/item), high use-decreasing (12.3% at 4–5 h/day/item) and low use-increasing (7.3% at 3 to nearly 5 h/day).^33^ Two studies found that depression predicts social media use^33,67^ and reduces perception of support.^51^ Specifically, Twitter use may be associated with depressive thoughts and symptoms, but only for people with low initial levels of in-person social support, and conveying positive sentiment helped to reduce depressive thoughts and feelings irrespective of people's level of in-person social support.^23^ Depressive signals observed in Tweets may predict future depression.^76^ Instagram browsing was associated with increases in depressed mood in adolescents.^42^

Type of media use is important, since hours spent on social media and internet use were more strongly associated with self-harm behaviours, depressive symptoms, low life satisfaction and low self-esteem than hours spent electronic gaming and watching television.^12^ In addition, girls generally demonstrated stronger associations between screen media time and mental health indicators than boys (e.g. heavy internet users were 166% more likely to have clinically relevant levels of depressive symptoms than low users for girls, compared with 75% more likely for boys). A cross-sectional study showed that cortisol systemic output was positively associated with Facebook network size and negatively associated with Facebook peer interactions.^50^

Studies of anxiety disorders are similar to findings in depression studies, with social anxiety symptoms mediated by spending more time on Facebook and passively using Facebook (i.e. viewing other's profiles without interacting).^62^ In a study with three focus groups of those with anxiety disorders, six themes emerged: seeking approval, fearing judgement, escalating interpersonal issues, wanting privacy, negotiating self and social identity and connecting and disconnecting.^41^ A qualitative study revealed three types of negative use, including ‘oversharing’ (frequent updates or too much personal information), ‘stressed posting’ (sharing negative updates) and encountering ‘triggering posts’.^46^ Both social anxiety and need for social assurance had a significant positive association with problematic use of Facebook^41,54^ or ‘fear of missing out’ (FOMO).^24,39^

### Clinical challenges like suicide, cyberbullying, sexting and other behaviours

The review found 34 studies on clinical challenges such as cyberbullying, sexting and posts on suicide (Supplementary Table 2). The primary populations were children (*n* = 1), adolescent (*n* = 15) and young adults (*n* = 14, with 3 for college students), with a mean age of 18 (median 17.9) years. The study populations were diverse in terms of ethnicity, but were predominately White and 15 studies were ≥50% female. The mean sample size was 34934.5 (median 524). The most common social media types studied were Facebook (*n* = 10), Twitter (*n* = 8), Instagram (*n* = 5), YouTube (*n* = 3) and MySpace (*n* = 2).

Excessive social media use, depression, suicide and school burn-out appear strongly related.^103,107,109,115^ One longitudinal study found that, compared with matched non-suicide-related Twitter posts, suicide-related posts were characterised by a higher word count, increased use of first-person pronouns and more references to death.^103^ In this study, emotional engagement, school burn-out and depression contributed to excessive social media use. Similarly, students with burn-out are at higher risk for depression and excessive social media use. Excessive social media use leads to school burn-out and school burn-out leads to excessive social media use. Individuals who were suicidal felt significantly less belongingness and significantly higher burdensomeness; they also use a higher proportion of achievement-related words and appear protective. Studies have compared artificial intelligence/machine learning to self-report measures to evaluate risk of suicide,^107^ para-suicidal events,^109^ suicide-related Tweets^112^ and other behaviors.^115^ Machine learning can easily differentiate people who are at high suicidal risk from those who are not (linguistic inquiry and word count, decision tree and cross-validation analyses).^107^ Machine-learning algorithms accurately identify the clinically significant suicidal rate in 92% of cases (sensitivity: 53%, specificity: 97%, positive predictive value: 75%, negative predictive value: 93%); a higher proportion of achievement-related words appears protective. For a single point of performance for comparison, artificial intelligence/machine learning had roughly 10% false alarms, but correctly identified about 70% of those who will attempt suicide.^109^

The relationship of depression, self-esteem and cyberbullying has been evaluated. A study of 8- to 13-year-olds evaluated whether cybervictimisation is prospectively related to negative self-cognitions and depressive symptoms beyond other types of victimisation.^110^ The majority of participants reported experiencing at least some degree of peer victimisation at either wave 1 or wave 2 (physical: 68.1%, relational: 89.8%, verbal: 87.9%, property related: 65.8%, cyber: 63.1%). Of note, 16.1% of participants obtained raw scores >75 on the Reynolds Adolescent Depression Scale – Version 2 (RADS-2), and 8.1% obtained scores >82 (signifying mild and moderate depression, respectively). Victimisation was correlated with negative cognition and depressive symptoms; it predicted depressive symptoms; age and gender were not predictors of cybervictimisation or depression. Depression is associated with problematic social media use and indirectly predicted cyberbullying perpetration (associations were weak). Another study found that problematic social media use is weakly correlated with depression (*r* = 0.22), gender (*r* = −0.15), age (*r* = −0.13) and self-esteem (*r* = −0.11).^95^ Experiences of LGBTQ participants included both help for coping and cyberbullying leading to depression, stress and suicidal ideation.^97^

Bystander responses to suicidal behaviour and cyberbullying are in sharp contrast. Only 33.6% of participants left a positive, supportive comment on at least one of two suicide posts. Content severity, experience with a loved one's suicide attempts and use of Facebook to meet people were predictive of providing positive comments.^94^ Positive bystander responses (PBRs) were higher in cyberbullying than traditional bullying incidents.^154^ Females exhibited more PBRs across both types of bullying. Bullying severity affected PBRs, in that PBRs increased across mild, moderate and severe incidents, consistent across traditional bullying and cyberbullying. PBRs related to cyberbullying included (a) seek help from a teacher or parent, (b) seek help from a peer or friend, (3) direct intervention and (d) providing comfort or emotional support.

Provider access to a patient's social media could assist in identifying suicidal ideation and/or acts, since patients fail to disclose risk factors to physicians; however, there are ethical and privacy concerns about searching a patient's social media platforms.^100^

### Health behaviour and well-being topics

There were 28 studies on health behaviour and well-being (Supplementary Table 3). The primary populations were adolescents,^8^ college students^14^ and young adults.^6^ The study populations were diverse in terms of ethnicity, but were predominately White and 19 studies were ≥50% female. The mean sample size was 1558.8 (median 15.8). The most common social media types studied were Facebook,^15^ Twitter (*n* = 2), Instagram (*n* = 1), YouTube (*n* = 1) and MySpace (*n* = 1). The most study population or disorder was depression (8) or anxiety (6).

Of the longitudinal studies, one found that a group deactivated from Facebook for 4 weeks showed small increases in well-being, but no changes in loneliness, compared with a usual use group.^124^ Another study over 2 months examined internalising symptoms (e.g. depression, anxiety and loneliness) related to the content of their Facebook communication and the responses they received from peers.^135^ The mean number of posts was 60.2 overall (88 for girls and 37 for boys). For girls, internalising symptoms predicted negative affect, somatic complaints and eliciting support; they also predicted receiving more peer comments expressing negative affect and peer responses offering support. A study over 9 months evaluated how social media activity affected individual social communication skill and self-esteem.^146^ Active social media use (i.e. directed, person-to-person exchanges) increases bonding and bridging social capital and decreases loneliness; passive use does not.

Cross-sectional studies of teenagers examined psychological well-being and differences between girls and boys in use of technologies,^12^ screen time ^27^^,125,126^ and social networking services (SNS).^142^ The study found that adolescent girls spent more time on smartphones, social media, texting, general computer use and online, and boys spent more time gaming and on electronic devices in general.^12^ Associations between moderate or heavy digital media use and low psychological well-being/mental health issues were generally larger for girls than for boys. For both girls and boys, heavy users (≥5 h) often rated twice as likely to experience well-being and mental health issues (e.g. risk factors for suicide) as low users. Also important was that the time 12th graders spent online doubled between 2006 and 2016; girls tend to spend more time in friendship dyads and boys in groups, and girls focus more on social relationships and popularity. A study of SNS and social self-concept, self-esteem and depressed mood found that the association between having an SNS and these negative indicators is more common with female youth; overall, frequency of SNS use is a positive predictor of social self-concept.^142^

With regard to college students, studies examined the relationship of social medial with well-being,^128^ FOMO,^130^ attachment, social capital^130^ and social closeness based on activity.^139^ Social media use is not associated with mental health problems, nor is emotional regulation; however, emotional regulation is associated with perceived stress and perceived stress is associated with mental health problems.^128^ Social media use does not indirectly predict mental health problems as mediated by perceived stress or emotional regulation. Social media use may indicate challenges with mental health issues or be a way of dealing with difficult emotions. When attachment theory was used to explore individuals’ attachment orientations towards Facebook use related to online and offline social capital, a secure attachment was positively associated with online bonding, bridging and all capital, and offline bridging capital; an avoidant attachment was negatively associated with online bonding capital.^138^ Anxious–ambivalent attachment had a direct association with online bonding capital and an indirect effect on all capital through Facebook. Users in the study on social closeness spent 7.82 min consuming content and 3.13 min on participation.^139^ Interacting with others on social media (e.g. commenting on updates) helps users feel closer to other people and this predicts positive emotional states after Facebook use. A study on FOMO involved two groups (10 min/day versus usual use), and both showed decreases in anxiety and FOMO; only the experimental group showed additional decreases in loneliness and depression.^130^ Moderation helps with mood and loneliness, and reduces anxiety and FOMO.

In a study on giving up Facebook, pre- and post-evaluation of perceived stress and well-being was measured by salivary cortisol between 14.00 and 17.00 h; those using Facebook had lower cortisol levels, less perceived stress, decreased life satisfaction and lower social loneliness on the Social and Emotional Loneliness Scale for Adults.^131^ One study examined that a user's activities on Twitter estimate a depressive tendency, based on a medium positive correlation (*r* = 0.45) between the Zung Self-Rating Depression Scale and the model estimations of potentially meaningful words (≤20).^146^ Although a total of 99 words had absolute values of correlation coefficients with Zung scores >0.4, the highest scores were associated with the following words: even if, very, workplace, hopeless, disappear, too much, sickness, bad and hospital.

### Implications for clinicians and researchers across clinical populations, problems and well-being

Findings of this scoping review inform approaches by providers, families and teachers when working with social media in children, adolescents and young adults (Table 1). To understand how technology affects the lives of adolescents and emergent adults, it is necessary to engage them in a conversation, share ideas and be available to help with problems. As many young people (and adults) may consider the internet their ‘lifeline’ to social engagement, consideration of the problematic aspects of internet use may be met with reluctance.^6,12,96,156^ Exploring beliefs, norms, values, cultural and language factors, and the meaning of technology to the individual, is integral to understanding and meeting the needs of each patient.^16,23,24,132^ For providers, the value of forming and maintaining a trusting, therapeutic alliance with youth cannot be overstated, as quality care depends on patient–provider engagement, open and honest communication and shared decision-making for treatment.^11,96,157^

An accurate assessment or history is needed of online activities and associated health and risk factors. Internet use may be healthy or problematic, and this continuum may be explored with youth and parents via non-judgemental questioning to clarify the types and extent of technology used (Table 1).^4,5,17,156^ Assessment is enhanced with multiple informants: parents, significant others, schools, primary care providers and/or others that know the youth well.^156,157^ How they use their time, what they enjoy, how they want others to view them, awareness/use of privacy settings and proneness to risky behaviours is a snapshot of esteem and quality of relationships.^157–159^

Providers, families and others need an approach to promote healthy use of social media and prevent problematic social media behaviours. Data on the relationship of social media use and its impact on behaviour – association, mediation or causation – and clinical interventions are limited.^4,5,9,14,158^ Nonetheless, positive family/home life, good engagement, supervision and other approaches may reduce risk of risky or dangerous behaviour.^4,24,38,156^ A shared understanding is needed about healthy versus problematic use, how to monitor use and blending social media with alternative activities to meet emotional needs. Individual, peer/group and family education and therapy is often helpful. Motivational interviewing techniques may help co-construct a plan that meshes with values, with parent and provider input.^3,24,156^

## Discussion

This scoping review provides an update to past reviews on evaluation, interventions and outcomes of social media related to clinical populations (e.g. mood and anxiety disorders), clinical challenges (e.g. suicide, cyberbullying) and health behaviour and psychological well-being in youth.^11,12,14–16,150^ This scoping review cast a much broader net and shows how substantial data can to contribute to diagnosis, monitor symptoms and collect ecologically rich behavioural data as a foundation for future interventions. Of 140 studies reviewed, longitudinal design,^19^ comparison groups^20^ and randomised controlled trials^3^ were uncommon, resulting in association (*n* = 120; 85.7%), mediating (*n* = 16; 11.4%) and causal (*n* = 4; 2.9%) relationships between social media and behavioural health issues. Specifically, the review found that social media use of >3 h appears to be associated with increased depression and anxiety, and passive browsing of social media appears to be associated with depression/anxiety compared with purposeful, positive and active engagement; more research is needed to verify these findings. Girls/young women are more likely to be disproportionately affected by depression/anxiety with regards to social media, which is potentially mediated by the type of interaction, whereas boys/young men have more difficult experiences with gaming. However, positive social support inside/outside of social media is protective (Supplementary Tables 1–3). Some studies have overlooked the impact of equity, diversity and inclusion related to social media use, and care is needed so that technology does not inadvertently contribute to inequity and other injustices. Any of the many dimensions of diversity or differences (e.g. culture, ethnicity, race, religion, sexual orientation, gender identity, language, nationality, immigration status, socioeconomic status, geography) could affect evaluation and intervention.

Research into social media is moving towards standardised methods, interventions and evaluation measures. Studies are limited or have not looked at key issues, such as (a) sociodemographics and health, digital and language literacy; (b) clinical population state or trait; (c) passive consumption, broadcasting and directed purposeful or active engagement/communication; (d) quality of assessment measures (e.g. standardised, clinician/provider-administered instruments or structured assessments rather than self-report questionnaires without confirmation, verification, observation and corroboration); (e) temporal dimensions of symptoms and assessment; and (f) longitudinal design and comparison groups. More information related to equity, diversity and inclusion for the populations using social media, their families and the clinicians involved with assessment and care is needed to evaluate the impact of differences, cultural safety and humility and potential interventions.^160^ This could include, but is not limited to, culture, ethnicity, race, religion, sexual orientation, gender identity, language, nationality, immigration status, socioeconomic status, spirituality, disability status, education, clinical diagnoses and geography. Implementation/ effectiveness designs – with longitudinal, quality of life and other dimensions – are also suggested,^157^ if well-anchored to health improvement.^161^ Data from existing empirical foundations, hierarchical evaluation systems and statistical analyses for multiple comparisons and un/adjusted analyses are needed.^157,161,162^

Research into social media could be helped by other advances in artificial intelligence, informatics and cognitive computing methods. These advance data processing, stratify risk (e.g. suicide) and predict future negative outcomes with longitudinal correlation, predict biomarkers/digital phenotypes (e.g. depression during and after pregnancy) and allow patients or providers to intervene for mood^65,76^ and suicide.^107,109,112,115,163^ Challenging issues include unique populations (e.g. culture, youth, college), the trade-off of privacy versus suicide detection and comparing artificial intelligence approaches with traditional methods. Social media, like wearable sensors, is transforming care by moving from manual transfer of subjective self-reported information during a patient visit to an integrated, longitudinal, minimally intrusive and interactive sharing of data based on the ecology of a person in their natural setting.^164,165^ Artificial intelligence inferential techniques (i.e. applied or performing functions similar to human thinking and analysis) have high predictive power and are reusable; suicide hotlines and face-to-face evaluations are effective methods for suicide intervention, but depend on action by the person with suicidal ideation.

Providers, parents/families and healthcare systems are facing challenges with social media, partly related to how youth live and how their developing brains are shaped by peers and the pervasive influence of technology.^156^ There are a range of behaviours across teenagers, adolescents and other age groups, and so a behaviour may be normal for one group and not for another; a behaviour may be healthy or problematic, depending on age. Families, teachers and providers can use data to engage youth with non-judgemental questioning about social media use, use preventive/risk factors for making decisions and, most importantly, stay as close as possible to their young loved ones who may be at risk for hurting themselves – while privacy is important on one hand, notification of families, clinicians and others who could help them may be helpful. Resources are also available from the American Academy of Pediatrics’ Media and Communication Toolkit and Family Media Use Plan,^158^ and other agencies.^166^ Competencies for social media, mobile health, wearable sensors and other asynchronous technologies^157,159^ include suggestions for training programmes (undergraduate/medical student, graduate/resident). These also address professional development of faculty and institutional change of health systems or academic centres to integrate video^167^ and asynchronous technologies.^157^

Scoping reviews appear more helpful than other types of reviews for evaluating the broad context, asking questions of the literature and generating questions, approaches, questions and methodologies for current and target states of research.^168^ There are limitations to this scoping review. First, a small team conducted the study selection and review, with only one reviewer screening all titles and abstracts. Second, a modified content analysis with thematic analysis components was presented, rather than a quantitative/numerical analysis of the extent and nature of the studies. Similarly, we categorised data into clinical disorders, but a different framework that looks at health from a functional perspective may have been a better option, such as the health continuum (from poor health/illness/languishing to good health/positive health/flourishing). Third, a quality evaluation tool was not used, partly because the diversity of study methodologies, duration and data collection make a thorough integrated review challenging, using a systematic quality evaluation system or the equivalent of a quantitative meta-analysis. In addition, a measure of risk of bias was not used, and is suggested when applicable and possible. There is also an inherent bias in studies of youth populations published in peer-reviewed literature. Cross-sectional studies of associations with multiple factors in applied rather than controlled settings have limitations. Fourth, the review does not cover all of the potentially relevant psychological well-being, stress and related life dimensions of youth. Fifth, this study did not assess if age or other sociodemographic characteristics were associated with or predicted types of social media use; furthermore, future studies and reviews may take the literature further by distinguishing between populations aged ≤17 years and those aged 18–25 years, as well as not extending this to 30 years of age. Sixth, broader input for consensus across organisations could have been helpful, and a qualitative, small-group interview approach with experts, using a semi-structured guide, could have discovered more information. Seventh, the review falls short of covering all psychiatric disorders (e.g. bipolar disorder, schizophrenia, developmental and other childhood disorders). Eighth, the review has some specific findings, yet points out generalised themes and questions; it is not a conclusive data analysis like a systematic review. Lastly, it is important to recognise the digital divide in social media use across different youth and sociodemographic populations, particularly for low-income, equity-seeking and deserving populations and populations in Latin America, Asia, Africa and Oceania.

In conclusion, research is moving forward on evaluation, intervention, monitoring and outcomes of social media use in youth related to clinical disorders, challenges like suicide and cyberbullying, and psychological well-being. Families, teachers and providers can use current data to engage youth with non-judgemental questioning about social media use and be aware of preventive/risk factors. Longitudinal comparison designs, effectiveness approaches, artificial intelligence and biomarking/digital phenotyping may provide a foundation for future interventions to examine causal relationships between social media use and behavioural health. Research opportunities and challenges can be broadly organised into the following categories: clinical outcomes from a functional perspective on a health continuum; diverse youth and sociodemographic populations, with age stratification by consensus, if possible (e.g. early adulthood to age 25, 30 or 34 years); methodology, models and data analytic approaches; development of consensus by ‘youth experts’ to provide input on the results and suggest youth-led and other intervention initiatives; study of human-computer-human interaction and privacy issues that inform policy. Whether effectiveness research on social media use can lead to better overall health outcomes and reduced disease burden is still unknown. Analysing large amounts of data will require close collaboration between partners from diverse areas of expertise, such as researchers, providers, statisticians, software developers and engineers. Health systems need to explore competencies for providers to place the person's/patient's needs first and embrace social media technology within healthcare reform, and this will require adjustment of clinical, training, professional development and administrative missions and workflow.

## Supporting information

Hilty et al. supplementary material Hilty et al. supplementary material

## Data Availability

The authors confirm that the data supporting the findings of this study are available within the article and its supplementary materials.
