# Supplementary material for: A scoping review of social media in child, adolescents and young adults: research findings in depression, anxiety and other clinical challenges
Source: BJPsych Open. 2023 Aug 11;9(5):e152. doi: 10.1192/bjo.2023.523 (PMC10594088; doi:10.1192/bjo.2023.523)
Supplement: Hilty et al. supplementary material [file S2056472423005239sup001.docx]

Supplementary Table 1 Child, adolescent and young adult social media research study outcomes for clinical populations and disorders

| # | STUDY | *N* | POPULATION | OBJECTIVE | DESIGN/METHODS/ MEASURES | OUTCOMES |
| --- | --- | --- | --- | --- | --- | --- |
| 1 | Akkin Gurbuz et al 2020^17^ | 374 | Adolescents, 13–18; Turkey | Explore relationship of SM use to behavioral problems | Cross-sectional Measure: Strengths and Difficulties Questionnaire; Online Social Networking Survey | Increased time spent on SM was associated with increased behavioral problems (*P* < 0.001) and peer problems (*P* = 0.003). |
| 2 | Barthorpe et al 2020^18^ | 4032 | Adolescents, 13–15; 55% female; UK | Explore associations between SM, screen-time and key MH outcomes like self-harm in the past year, depressive symptoms, and self-esteem | Cross-sectional of Twitter, FB, BBM, Snapchat within the Millennium Cohort Study Measures: time use diaries; SMFQ depressive symptoms; and shortened RSEQ esteem | Self-harm 14.7% in the past year (20.2% females; 6.5% males)  Increased time spent on SM was associated with an increased risk of self-harm (adjusted OR per 30 min increase in weekday use: 1.13) and depression (adjusted *B*= 0.36) and lower levels of self-esteem (adjusted *B*= −0.12) in females Findings were similar for weekday and weekend use |
| 3 | Coyne et al 2020^19^ | 500 | Adolescents, from 13 to 20 yrs; 51.6% female; 65.4% European Am, 12.2% AA; US | Examine the association between time spent using SM and depression and anxiety at the intra-individual level | 8-year longitudinal study: annual questionnaire Measures: SM use from none to 8+ h; CES-DC; SCIA | Overall, SM use increased from 30–60 min/day to 2 h/day Mean depressive levels increased throughout adolescence Moderate to large sex differences (e.g., girls reporting higher depressive symptoms and anxiety) Time spent on SM was not associated with increased MH issues |
| 4 | Nereim et al 2020^20^ | 55 | Adolescents, 14–19, mean 17.4 yrs; 67.3% female; 27.8% White, 29.6% Black, 27.8% Hispanic; US | Evaluates whether race/ethnicity or baseline anxiety and/ or depression levels impact momentary affect with passive *v*. active SM use | Exploratory 7-day EMA with 5 daily surveys via smartphone Measures: PHQ; SAS-A; and PANAS | Mean PHQ-9: White (14.0) scoring higher than Hispanic (9.2) Mean SAS-A: White (57.3) scoring higher than Black (46.4) SM use frequency was unrelated to PHQ-9 or SAS-A and did not differ by race. Blacks reported a higher % passive use (48.5%) than Hispanics (12.4%), which is more strongly associated with depression |
| 5 | Twenge et al 2020^21^ | 11 427 | Adolescents, mean 13.8 yrs; 50% female; UK | Examine if hrs/day spent on specific screen media activities is linked with 4 MH indicators: self-harm behavior, depressive symptoms, life satisfaction, and self-esteem | Longitudinal over 2 years Millennium Cohort Study Measures: SMFQ; RSES | SM use higher among girls and gaming higher among boys; 1/3 reported spending 5+ h /day on the Internet Hours spent on SM and Internet use were more strongly associated than gaming and TV watching with self-harm behaviors, depressive symptoms and low self-esteem Girls generally demonstrated stronger associations between screen media time and MH indicators like depression |
| 6 | Brunborg et al 2019^22^ | 763 | Adolescents, mean yrs 15.2; 53.9% female; Norway | Examined if time spent with active use was associated with changes in depression, conduct problems, and episodic heavy drinking | Longitudinal over 6-months Using first- differencing models (FD-models) | Average about 2.5 h per day and increased 0.39 h Time spent on SM at T1 was positively associated with depression (*r*= 0.31), conduct problems (*r*= 0.16), and episodic heavy drinking frequency (*r*= 0.22) at T1 – and correlated with increases in symptoms of depression (*r*= 0.11), conduct problems (*r*= 0.16), and episodic heavy drinking (*r*= 0.13) at T2 |
| 7 | Cole et al 2019^23^ | 308 | All ages/adults, 30 yrs; 52% female; 10.54% Black, 75.20% White, 6.36% Hispanic, 10.02% Asian Am; US | Examine if twitter use is associated with reduced depressive symptoms, and if in-person social support moderates this | Longitudinal over 4 months Used Amazon.com Mechanical Turk to recruit master workers Measures: PSS Scale; BDI-II; CTI (negative views) | Twitter use by those with low in-person social support interactions predicted positive change in depression-related outcomes over time Having a larger twitter social network and being more active in that network are especially helpful to people who have lower levels of in-person social support |
| 8 | Dempsey et al 2019^24^ | 296 | College students, mean 20.0 yrs and 95.8% 18–25; 57.6% female; 73.9% White, 12.4% Black; US | Examine if problematic FB use predicts depression, social anxiety and lower life satisfaction via rumination or FOMO; age and gender covariates | Cross-sectional Measures: FoMO scale; PHQ-9; RTSQ; and SWLS | Modeling results demonstrate that FOMO and rumination were signiﬁcantly related to PFU severity FB use frequency was related to problematic use severity. FoMO and rumination each mediated relations between social anxiety and problematic use severity |
| 9 | Jensen et al 2019^25^ | 2100 | Adolescents, 11–15 yrs; US | Examine if SM use predicts MH symptoms | Longitudinal over 14.4 yrs with 13 017 total observations over 5,270 study days EMA | Technology usage did not predict later MH symptoms; indeed, teens who reported sending more text messages over the study period reported feeling better (less depressed) than teens who texted less frequently |
| 10 | Ophir et al 2019^26^ | 248 | Adolescents, 13–18; yrs; 51% female; Hebrew-speakers; Israel | Explore predictive validity of references to personal explicit distress in FB Content: (a) Fun and news, (b) Commercials, (c) Belonging, d) Values and involvement, (e) Gothic and dark, and (f) Extreme and offensive | Cross-sectional 2 studies: 1, explicit references with 1168 posts over 3 mo; 2, data on depression, social rejection, and bullying Measures: BDI-II (9–10 mean); YSR (social problems); and PRQ (bullying) | Study 1: explicit distress rarely (12%) predicted depression Study 2: less explicit features of FB behavior (‘posts by others’, ‘check-ins’, ‘gothic and dark content’, ‘other people in pictures’, and ‘positive attitudes towards others’) predict social rejection and victimization of bullying Bullying associated with active FB ‘Your posts’ and ‘Fun and news’), as well as use of first person single pronouns |
| 11 | Orben, Dienlin et al 2019^27^ | 12 672 | Adolescents, 10–15 yr olds, in UK | Evaluate SM use and depression and anxiety Computational method with theoretically grounded analysis to reduce false +s | Longitudinal | Negative association between SM use and life satisfaction; depression and anxiety SM is not a strong predictor of life satisfaction, though it was a predictor of slightly decreased life satisfaction across all domains for females |
| 12 | Riehm et al 2019^28^ | 6595 | Adolescents, 12–15 yrs forward; 48.7% female; US | Assess whether time spent is prospectively associated with internalizing (i.e., depression, anxiety) and externalizing problems FB, Google Plus, YouTube, MySpace, Linkedin, Twitter, Tumblr, Instagram, Pinterest, or Snapchat | Longitudinal cohort study with annual data at 1, 2 and 3 yrs; Population Assessment of Tobacco and Health study Time intervals: none, <30 min, >30 min to <3 h, >3 h to <6 h, and >6 h Measures: GAIN-SS | Problems: 611 (9.1%) reported internalizing, 885 (14.0%) reported externalizing (e.g., bullying), 1169 (17.7%) reported both and 3930 (59.3%) none/low SM use: 1125 adolescents (16.8%) none, 2082 (31.8%) ≤30 min, 2000 (30.7%) 30 min-3 h, 817 (12.3%) 3–6 h and 571 (8.4%) 6+ h/day Association between SM use (3+ h *v*. none) and internalizing and combined internalizing/externalizing problems 1 yr later, controlling for psychopathology and alcohol/marijuana use |
| 13 | Xie et al 2019^29^ | 526 | College students, mean 24.2 yrs; 59% female; 68.4%, White, 12.7% Asian. 7.0% Hispanic, and 6.3% Black; US | Examine FB addiction and state anxiety without it | Cross-sectional survey Measures: FIS intensity; BFAS addiction; STAI trait; and SF-SAS state anxiety | FB use intensity predicts FB addiction and state anxiety. Its use for broadcasting positively predicts addiction and state anxiety Trait anxiety positively predicts FB addiction and state anxiety Gender interacts with trait anxiety and jointly predicts FB addiction |
| 14 | Yuen et al 2019^30^ | 312 | College students, 18–25 mean 18.8 yrs; 79% female; 79.6% Caucasian, 7.1% Black, 6.7% Multiracial, 4.2% Asian/Pacific Islander; US | Examine how FB activity affects mood comparing: (1) browse the Internet, passively browse others’ profiles, actively communicate with others via messages/posts, or updating personal profile | Quasi-experimental, randomized groups for 20 min activities:  Measures: PANAS; FES; and perceived meaningfulness of activity | FB use several times a day (51%) or weekly (31%); visited other SM sites several times a day: Instagram (77.6%), Twitter (44.9%), YouTube (24%), Tumblr (9.0%), Pinterest (8.3%), Google Plus (7.7%), LinkedIn (0.6%), and Reddit (0.6%) Passive FB use led to worse mood than when using Internet Perceptions of meaningfulness, but not feelings of envy, mediated the relationship between online activity and mood |
| 15 | Bayer et al 2018^31^ | 154 | College students, mean 20.3 yrs; 67% female; US | Evaluate SM use and depression and anxiety | Longitudinal over 2 wks | Negative association; necessary to differentiate the emotional and social outcomes of SM use FB activities predicted no changes in aggregate mood over 2 weeks, despite showing positive relationships to bridging social capital during same time |
| 16 | Berryman et al 2018^32^ | 467 | College students, mean yrs 19.7; 71.7% female; 60.2% White, 16.5% Latino/a or Hispanic, 10.1% Black, 6.6% Asian Am; US | Examine the relationship between SM use and MH: general symptoms, suicidal ideation, loneliness, social anxiety and decreased empathy | Cross-sectional Measures: time spent in hrs; vaguebooking 3 questions; SMUIS importance of use; BSI-18; PSS; Parent-Child Conflict via PIY; BHPS*;* NTB; UCLA Loneliness Scale; IRI; and M-C SDS-SF | SM use or importance was not predictive of impaired MH functioning, but vaguebooking (posting unclear but alarming sounding posts to get attention) predicted SI and loneliness Social desirability and support were consistently associated with less reporting of negative symptoms Perceived parent/child conflict predicted MH symptoms, suicidal thoughts and loneliness. Need to belong was associated with most negative outcomes |
| 17 | Houghton et al 2018^33^ | 1749 | Adolescents, 10–17 yrs, 49% female; Australia | Identify trajectories of depressive symptoms and bi-directional associations with screen use time Weekday and weekend time use was weighted in the ratio 5:2 | Prospective longitudinal: six waves of data over two years Self-reported screen time via SM, gaming, web browsing, and TV/passive; given 20 different screen use options Measures: SBMUS (screen time); CDI-2 | Screen use to depressive symptoms trajectories were: low-stable (80%; average 3–4 hr/day/item); high-decreasing (12.3%); average 4–5 hr/day/item), and low-increasing (7.3%; 3 to nearly 5 hr/day) were identified. (Average means the low-increasing had trajectories increased across all four types) Substantial bi-directional associations were observed; a Random Intercept Cross Lagged Panel Model revealed no consistent support for a longitudinal association |
| 18 | Niu et al 2018^34^ | 764 | Adolescents, 12–18 yrs; 46.8% female; China | Explore relationship between SM (Qzone) use and depression, mediated by negative social comparison and self-esteem | Cross-sectional | Negative social comparison mediated the relationship between Qzone use and depression Qzone use was less strongly associated with negative social comparison at higher levels of self-esteem |
| 19 | Reineke et al 2018^35^ | 818 | Early and middle adolescents, mean 14.1 yrs; Germany | Evaluate SM use, trait procrastination and psychological functioning (i.e., stress, sleep quality); depression and anxiety | Longitudinal | Negative association except trait procrastination was positively related to Internet multitasking and uncontrolled Internet use Internet multitasking partially mediates the association between trait procrastination and psychological functioning (i.e., stress, sleep quality, mood, anxiety) |
| 20 | Twenge et al 2018^36^ | 506 820 | Grades 8 -12, mean 16.1 yrs; US | Report suicide deaths for those ages 13–18, depressive symptoms, suicide­related outcomes, and suicide rates | 2010–15 period Compare new media (SM, SPs) *v*. non-screen activities (e.g., in person social, sports/exercise, homework) Measures: MTF; and YRBSS | Since 2010, iGens have spent more time on new media screen *v*. less time on non-screen activities and television Depressive symptoms, suicide­related outcomes, and suicide deaths all rose during the 2010s (after a stable period) More time on new media linked with MH issues *r* = 0.06 (overall, girls but not boys) *v*. non-screen activities |
| 21 | Akkin Gürbüz et al 2017^37^ | 108 | Adolescents, mean 15.3 yrs; Turkey | Evaluate the SM habits (time spent) of depressed adolescents and relationship between depression and disclosure | Cross-sectional comparing depressed *v*. non-depressed Measures: K-SADS-PL; CDI; SAS; and Social Network Use Questionnaire | Time spent on the Internet and on SM was significantly higher among depressed than non-depressed adolescents The intensity of the depression sharing was significantly higher in the depressed group |
| 22 | Bányai et al 2017^38^ | 5961 | Adolescents, mean 16.6 yrs; 51% female; Hungary | Estimate SM problematic use: no, low and high-risk stratification  CES-D BSMAS | Cross-sectional rating of last 12 months European School Survey Project on Alcohol and Other Drugs (ESPAD) | 23.1 h/week (s.d. = 15.6) 4.5% over the last 12 months belonged to the at-risk group, and they reported low self-esteem, high level of depression symptoms, and elevated use; had the highest withdrawal score BSMAS: specificity 99% and the sensitivity is 83% |
| 23 | Barry et al 2017^39^ | 113 | Adolescents (and their parents) in high school; 14–17 yrs; mean 15.3 yrs, 49% female; 82% White; US | Determine the relations between use (i.e., frequency) and various indices of MH and psychosocial functioning | Cross-sectional Measure: DSM-5 Checklist | Parent and adolescent reports of time spent are correlated, *r =*0*.*74 Male adolescents > females report being friends with parents; age was positively correlated with parent- *r =*0*.*30 and adolescent-reported *r =*0*.*30 time since use initiated The number of adolescents’ accounts were moderately correlated with parent-reported DSM-5 inattention, hyperactivity/ impulsivity, ODD, anxiety, depression, FOMO and loneliness |
| 24 | Brunborg et al 2017^40^ | 851 | Middle and high school students, mean 14.1 yrs, 53.9% female; Norway | Examine if SM time spent is associated with depression, conduct problems, and episodic heavy drinking | Cross-sectional  Measures: PHQ-9; MacArthur Scale of Subjective Social Status; Barratt Impulsiveness Scale– Brief; Brief Sensation Seeking Scale; and Peer Relationship Problem scale | Greater amount of time spent was associated with greater likelihood of episodic heavy drinking among adolescents, even after adjusting for school grade, impulsivity, sensation seeking, symptoms of depression, and peer relationship problems |
| 25 | Calancie et al 2017^41^ | 8 | Adolescents, 13–18, mean yrs 15.5, ‘high risk’ area (i.e., disadvantaged communities); Canada | Use qualitative analysis to explore anxiety disorder patients’ experiences with FB | Longitudinal 3 focus group discussions attended by a total of 8 adolescents (4 females and 4 males) | 6 themes: seeking approval; fearing judgment; escalating interpersonal issues; wanting privacy; negotiating self and social identity; and connecting and disconnecting Fear of receiving negative comments online led to less posting of comments and photographs Initial positive emotions from a ‘like’ often felt ‘false’, ‘unreal’ and ‘fleeting’ |
| 26 | Frison et al 2017^42^ | 671 | Adolescents, 12–19, mean 14.9 yrs; 61% females, Belgium | Evaluate SM use – mainly Instagram (browsing, posting, and liking on Instagram) and depression and anxiety Frequency | Longitudinal over 6 months with structural equation modeling Measures: CES-DC; and SEM | Instagram *browsing* at T1 was related to increases in depressed mood at T2, but were not related to depressed mood at T2 Depressed mood at T1 was related to increases in Instagram *posting* at T2; depressed mood at T1, however, did not predict Instagram *browsing* and liking at T2 Similar for boys and girls Predictors explained 28% of the variance in depressed mood, and 35% of Instagram posting, 20% of browsing and 24% of liking |
| 27 | Kokkinos et al 2017^43^ | 240 | Adolescents and young adults, mean 21.5 yrs; Greece | Investigate the associations between risky FB lifestyles, individual differences, risk factors, and victimization | Cross-sectional Measures: FB victimization (cyberbullying); and PHQ-9 | Personality variables and risky FB lifestyle (e.g., indiscreet content, time spent and friends) were linked to victimization Depression was the strongest predictor - followed by indiscreet content and self-disclosure - of victimization through FB |
| 28 | Lee et al 2017^44^ | 4920 | Youth, 13–21; Singapore | Improve the social cognitive model by understanding both external and personal-level antecedent factors | Cross-sectional survey | Depression was positively associated with self-reactive outcome expectation and deficient self-regulation Positive relationship with father (not mother) is negatively associated with dependence on SM for identity formation |
| 29 | Oberst et al 2017^45^ | 1468 | Adolescents, mean 16.6 yrs; 74% female; Spanish-speaking Latin-Am countries | Analyze the role of FOMO and intensity of SNS use link psychopathological symptoms and negative consequences | Cross-sectional with structural equation modeling Measures: HADS; SNI; FOMO scale; and CERM (negative consequences) | FB (99.3%), WhatsApp (59.0%), Twitter (44.2%), Instagram (35.0%) and Google plus (35.1%). Both FOMO and SNI mediate the link between psychopathology and CERM, but by different mechanisms: for girls, via feeling depressed; and for boys, via anxiety |
| 30 | Radovic et al 2017^46^ | 23 | Adolescents, 13–20 yrs, from adolescent health clinic; 78% female; US | Explore how SM use may influence and be influenced by psychological distress | Qualitative study (30–60 min semi-structured interviews in-person or via telephone | Both positive (searching for information and social connection) and negative use (risky behaviors, cyberbullying, and self-denigrating comparisons with others) Negative use: ‘oversharing’ (frequent updates or too much personal information), ‘stressed posting’ (negative updates), and encountering ‘triggering posts’ |
| 31 | Radovic et al 2017^46^ | 23 | Adolescents with depression and SI, 13–20, mean 16 yrs; 78% female; psychiatric clinic US | Explore in depressed patients how SM use may influence and be influenced by psychological stress | Cross-sectional, qualitative, semi-structured in-person or over telephone FB (10), Tumblr (4), Twitter (4), and Instagram (4) | Depressed adolescents had a much larger network of FB friends (460 *v*. 145 *v*. Pew, 2012) and were friends with strangers (55% *v*. 33% *v*. Pew, 2012) |
| 32 | Vernon et al 2017^47^ | 874 | Adolescents, mean 14.4 yrs; 59% female; Australia | Evaluate impact of use on mood and other health | Longitudinal cohort study Mood scale adapted from others | Increased investment of time used predicts depressed mood, partly due to sleep disruptions Both problematic social networking and sleep disruption underwent positive linear growth over time |
| 33 | Frison, Eggermont et al 2016A^48^ | 1886 | High school students. 12–19, mean 15.4 yrs; Belgium | Examined FB link of daily stress (i.e., school- and family-related), social support seeking, perceived social support and depressed mood | Cross-sectional with structural equation modeling Measures: frequency; stress via ASQ; perceived social support via MSPSS; and CES-DC | Means: daily stress 3.25, social support seeking 1.55, perceived social support 2.11 and depressed mood 1.58 Daily stress positively predicted only *girls’* depressed mood Daily stress positively predicted seeking of social support through FB: if perceived (i.e., received), it *decreased* depressed mood; if not perceived, it *increased* depressed mood |
| 34 | Frison, Subrahmanyam et al 2016B^49^ | 1621 | Adolescent 12–19, mean 14.7 yrs; 48% female; Belgium | Examines the reciprocal FB relationships between peer victimization, depression and life satisfaction; moderating role of gender, age, and perceived support | Short-term longitudinal (two-wave with 6 mo interval) Measures: time spent; SN-PEQ for FB; CES-DC; satisfaction with Life Scale; and MSPSS | Cross-lagged analyses indicated that peer victimization marginally predicted decreases in life satisfaction, and life satisfaction predicted decreases in peer victimization Depressive symptoms were a risk factor for peer victimization rather than an outcome. Support from friends protected against peer victimization |
| 35 | Morin-Majer et al 2016^50^ | 88 | Adolescents 12–17, mean 14.5 yrs; 53% female; Canada | Explore the associations between FB behaviors (use frequency, network size, self-presentation and peer-interaction) and basal levels of cortisol | Cross-sectional with 4 cortisol samples per day for two non-consecutive weekdays Measures: CDI (French-validated version); PSS Scale; Perceived Stress Scale; and RSEQ | Hierarchical regression showed cortisol systemic output was positively associated with the number of friends and negatively associated with peer-interaction; no associations were found among depressive symptoms, self-esteem and cortisol Number of friends is positively correlated, while peer-interaction is negatively associated, with cortisol levels |
| 36 | Park et al 2016^51^ | 61 | Youth, mean 19.9 yrs, 61% female, 28.8% Caucasian, 17.8% Asian, 8.2% Black; US | Examined how disclosing positive or negative information to those depressed on FB inﬂuences *perceived* *v*. *received* social support | Cross-sectional Modified SPS (12-item) for social support | If those depressed disclosed negative information, it was *positively* correlated with received social support and *negatively* correlated with perceived social support (*r*(56)¼-.37, *p*¼.005); higher depressive symptoms were associated received social support Depressive symptoms and positive disclosure were not correlated |
| 37 | Park et al 2016^51^ | 42 | Young adults, mean 26.8 yrs and controls 23 yrs; approximately 85% female and 70% Caucasian; US | Examine the social support on FB via disclosure by depressed and non-depressed individuals (i.e., perceptions of FB support) | Longitudinal: initial and 9-mo follow-up Measures: SCID; BDI-II; discrepancy index; and modified SPS | If those depressed disclosed negative information, it was *positively* correlated with received social support and *negatively* correlated with perceived support Discrepancy index (received *v*. perceived) was signiﬁcantly higher for depressed than non-depressed participants |
| 38 | Banjanin et al 2015^52^ | 336 | Adolescents in high school, mean 18 yrs; 66% female; Belgrade, Serbia | Evaluate relationship between use (i.e., time spent) and depression | Cross-sectional Measures: CES-DC; and Young Internet Addiction Test (IAT) | The average time spent was 3.11 h ± 2.82 h per day No relationship existed between depression, time spent and SM activities (e.g., number of FB friends) Internet addiction and depression correlated (*r*=  + 0.27) |
| 39 | Coppersmith et al 2015^53^ | NS | Adolescents and adults, mean female 21.2 and male 23.4 yrs; 75% English + tweets; US | To explore tweet (Twitter API) content with self-reported health sentences and language differences MH: ADHD, BD, BPD, depression, ED, GAD, OCD, PTSD, schizophrenia | Cross-sectional Compare language differences in 10 MH conditions LIWC, open vocabulary, CLMs with age- and gender-matched controls | Quantifiable signals exist for 10 MH conditions in messages with a precision range of 85% (GAD), 67% (ED), 63% (schizophrenia) and 48% (depression) CLMs are unique to condition, with cross-validation; cross-condition comparisons via new CLMs showed Pearson correlations for ED and GAD, PTSD and BP and schizophrenia and OCD |
| 40 | Lee-Won et al 2015^54^ | 243 | College students, 18–24 yrs; 71.6% female; 91.8%, white, 1.6% Black, 2.1% Asian, 1.2% Hispanic, 1.2% Native Am; US | Investigate if social anxiety and need for social assurance cause problematic use of FB Ranges of use:<10 min, 10–30 min, <1 h, 2, <3-and ≥3 h | Cross-sectional survey, 6-item social anxiety, 8-item need for social assurance and 4 of Big 5 personality traits (agreeableness, extraversion, conscientiousness, neuroticism) | Social anxiety and need for social assurance are positively associated with problematic use; the latter served as a significant moderator with 31.9% of the total variance Conscientiousness (*b*= –0.52, *b*= –0.13, *P*< 0.05) and amount of use (*b*= 1.54, *b*= 0.38, *P*< 0.001) are associated with problematic use only for those with medium to high levels of need for social assurance |
| 41 | Lup et al 2015^55^ | 116 | Youth, mean 24.8 yrs; 84% female; US | Tested association between Instagram use and depressive symptoms mediated by negative social comparison and amount of strangers followed | Cross-sectional with a theoretically grounded moderated meditation model Measures: SCRS; and CES-D | Instagram use was marginally positively associated with depressive symptoms, and positive social comparison was significantly negatively associated with depressive symptoms Amount of strangers moderated the associations of Instagram use with social comparison (significantly) and depressive symptoms (marginally) |
| 42 | McCloskey et al 2015^56^ | 633 | Youth, mean 21 yrs; 70% female, Caucasian 64.8%; US | Develop an empirically sound measure of social support (FMSS) and compare it to depression and QOL measures Exploratory factor analysis and retention analysis | Cross-sectional Measures: PHQ-9; FMSS; ISSB; MSPSS; and WHOQOL-BREF | FMSS assesses four factors of support (Perceived, Emotional, Negative, Received/ Instrumental); the Negative Support factor was most strongly related to both depression and QOL Elevations in the Emotional factor was associated with worse symptoms of depression and poorer psychological QOL Perceived social support was negatively associated with depression severity and positively associated with all domains of QOL; received social support was not significantly related to depression but was positively related to QOL Psychological, Social Relationships and Environment factors |
| 43 | Mitchell et al 2015^57^ | 174 | All ages, Twitter API, 2008-15, 3200 tweets; US | To use several NLP techniques to explore the language of schizophrenic users on Twitter | Lexicon, LDA, open vocabulary, CLMs, Brown clustering | CLM features were used to train models to classify users with and without schizophrenia; LDA outperformed LIWC. Of those with schizophrenia, other common diagnoses were depression (14), PTSD (8) and GAD (6) |
| 44 | Moberg and Anestis 2015^58^ | 305 | Youth, mean 20.6 yrs, 84% female; US | Evaluate if social networking negative experiences are associated with belongingness | Measures: time spent; and DASS-21 | Negative interactions on social networking sites significantly predicted thwarted belongingness; no predictors were associated with perceived burdensomeness after accounting for demographics, depression, and thwarted belongingness |
| 45 | Nesi and Prinstein 2015^59^ | 619 | Adolescents (12–17), 57% female; US | Examine use and depressive symptoms Short Mood and Feelings Questionnaire | Longitudinal cohort | Significant relationship was found between these online behaviors at baseline and depression 12 months later (technology-based social comparison and feedback-seeking time 1/depression time 2 *r* = 0.34) |
| 46 | Park et al 2015^60^ | 212 | Young adults, mean yrs 25.6, 24% females; Canada | To analyze the relationships between FB activities and the depression state using web application | Cross-sectional, 2 wks, with online and offline data integration Measures: CES-D; and BDI | CES-D score 21–60 (high probability of depression) for 42/212 Depressed pts: fewer interactions (e.g., 4.2 receiving likes *v*. controls); and posted at a higher rate particularly mid-day |
| 47 | Preotiuc-Pietro et al 2015^61^ | 1145 | Young adults, mean 25 yrs, 30 000 + *n*-grams, 1500 followers/person; US | To implement Twitter linear classifiers for self-disclosed illness to compare: (a) control *v*. depression; (b) control *v*. PTSD; and (c) depression *v*. PTSD | Use corpus metadata with textual and topic features, and standard linear classifiers (i.e. Logistic Regression and Linear SVM; 5 clustering types including LDA) | The combination of linear classifiers performed better than average classifiers at 80% and models achieve over *.*806 average precision (AP) for all three binary tasks; controls *v*. PTSD and controls *v*. depressed users easier than distinguishing PTSD from depressed users |
| 48 | Shaw et al 2015^62^ | 75 | Youth, mean 19.2 yrs, 55% female; US | Non-diagnosed social anxiety FB users completed questionnaires about psychological symptoms, FB usage and brooding | Cross-sectional Measures: FB Activity Measure; and CES-D | Greater social anxiety symptoms were associated with time spent and passive use Brooding mediated the relationship between passive use and social anxiety symptoms; an alternative model suggested that social anxiety mediated passive use and brooding |
| 49 | Tandoc Jr. et al 2015^63^ | 727 | College students, mean 19 yrs, 78% female; US | Uses the framework of social rank theory of depression to evaluate use, envy, and depression | FB may help positive self-presentation or a measure of social attractiveness, as a form of resource Measures: CES-D | Heavy FB users experienced stronger feelings of envy and more symptoms of depression, even when controlling for the effects of age and gender FB envy was the only signiﬁcant and positive predictor of depression |
| 50 | Davidson et al 2014^64^ | 336 | College students; 70% female; 80% White, 66% live on campus, and 72% in Greek life; US | Examine relationship of religiosity, social and FB-specific anxiety, role conflict and FB intensity | Cross-sectional Measures: FIS; SAS; RCS; 14 Likert FB anxiety and religious practice questions | Correlation analyses indicate a connection between FB use and anxiety, as well as a link between religiosity and anxiety Role Conflict correlates with FB Intensity, FB-specific anxiety, and social anxiety |
| 51 | De Choudhury et al 2014^65^ | 165 | Child-bearing, 20–39, mean 28 yrs; 16.7% depressed; US | Use AI/ML to explore several types of FB data to detect and predict postpartum depression and semi-structured interviews | Longitudinal: 50 wks pre-birth and 10 wks post-birth Measures: PHQ-9; social capital; and linguistic style  PHQ-9 | Postpartum depression was predicted by increased social isolation, decreased social capital (i.e., less activity, interaction) and fewer status updates 14.8%, media items 8.8% and reaching out to friends 30%; also with more highly volatile (i.e., higher entropy) activity 15% |
| 52 | Farquar et al 2014^66^ | 250 | College students, mean 19.9 yrs; 68% female; USA | Examine the impact of FB use on social anxiety and FB-specific anxiety | Cross-sectional Measures: FIS; SAS; RCS; 14 Likert FB anxiety and religious practice questions | Predictors included the intensity of use, role conflict experienced during use, user religiosity and user self-monitoring activities (which decreased FB-specific anxiety but increases general social anxiety) |
| 53 | Gamez-Guadix et al 2014^67^ | 951 | Adolescents, 13-17; 11% female; Spain | Examine SM use and depressive symptoms | Longitudinal cohort | A bidirectional relationship was found between depression and use: T1 predicted increase in the latter at T2, and vice versa |
| 54 | Király et al 2014^68^ | 4,875 | Adolescents, mean 16.4 yrs, 32% female; Hungary | Compare use in time spent and mood symptoms for problematic Internet use (PIU) and problematic online gaming (POG) | European School Survey Project on Alcohol and Other Drugs Project CES-D, RSES, (PIUQ-6) and POGQ-SF | Notable difference between the two problem behaviors in sex: POG more strongly associated with being male. Self-esteem had low effect sizes on both behaviors, while depressive symptoms were associated with both PIU and POG, affecting PIU slightly more |
| 55 | Labrague et al 2014^69^ | 76 | Nursing BSN students; 16–20 yrs; Philippines | Examined anxiety and depression on FB; 77.63% have more than 400 friends with an average 1.3 h/day | Cross-sectional Measures: FIS; and DASS | Prevalence rates of depression, anxiety, and stress among respondents were 28.95%, 48.68%, and 25% Time spent on correlated significantly with depression (*r* = 0.233, *P* = 0.041) and anxiety (*r* = 0.259, *P* = 0.023). |
| 56 | Lee et al 2014^70^ | 199 | Youth, college students (18–23), mean 19.9 38% female; US | Examine social comparison behavior: SCO; self-uncertainty; self-esteem; and frequency/time spent with expectation of response on FB | Personality characteristics includes social comparison orientation and self-esteem and self-consciousness Measures: Likert questions; and CES-D | SCO, self-uncertainty, and self-esteem correlate with social comparison frequency on FB 0.47, −0.54 and −0.29 respectively Negative feeling from comparison on FB associated with SCO and self-esteem; positive association of SC frequency and the frequency of having a negative feeling from comparison |
| 57 | Neira and Barber 2014^71^ | 1819 | Adolescents, 13–17 with mean 14.6 yrs; 55% female; 83.4% Caucasian, 7.2% Asian, 1.5% Black; Australia | Evaluate relationship between SM and life adjustment including self-esteem and mood on FB, MySpace and Bebo | Cross-sectional Measures: Depressed Mood Scale; frequency of use (never to several times/day); investment; and adjustment to life | No link of depression and frequency of use to social self‐concept *v*. non-users, while investment was associated with lower self‐esteem and more depressed mood Having a SM site was linked to more negative indicators for females than males |
| 58 | Schwartz et al 2014^72^ | 28 749 | All ages, mean yrs 24.8; 57% female; US | To build a FB predictive regression model to detect depression and compare it to a sentiment lexicon | Predict and characterize one’s degree of depression (*DDep*) based on language use (n-grams, LDA and lexica) | FB updates enabled distinguishing depressed users, with *r* = 0.149 Predictive models offered insights into seasonal affective disorder: *DDep* values, as predicted by language, were significantly higher in the winter months |
| 59 | Simoncic et al 2014^73^ | 237 | Youth (18–23), college, mean 18.8 yrs; 47% female; Black (5%), Asian Am (11%), Caucasian (77%), and other (7%); US | Examine any association between FB use and depressive symptoms as moderated by personality and sex | Cross-sectional Time: 30- to 60-min increments Measures: CES-D; FB Questionnaire; and individual factors and personality domains (i.e., extroversion and neuroticism) (NEO PI-R) | No direct association was found between FB use and depressive symptoms. However, for females with high neuroticism, more frequent FB use was associated with lower depressive symptoms Complex relationship between FB use and depressive symptomatology that appears to vary by sex and personality |
| 60 | Steers et al 2014^74^ | 180 | Varied 19–57, mean 24.4 yrs; 78% female; 17% Black, 17% Asian Am, 26% Hispanic, 32% White; US | Evaluate time spent on social comparisons (more) and depressive symptoms, and see if social comparisons mediate for depression | Cross-sectional Measures: CES-D; INCOM for FB for active and non-active users; and time spent (30 min-1 h, 1–2 h, 2–3 h, 3–4 h, and 4+ h) | Association between time spent on FB and depressive symptoms for both genders; making social comparisons mediated this in men only Depressed pts did not spend more time doing social comparisons |
| 61 | Steers et al 2014^74^ | 152 | Youth 18–32, mean 22.5 yrs; 62% female; 22% Asian Am, 31% Hispanic, 25% Caucasian, 15% Black; US | Evaluate FB logins and time spent on depressive symptoms and social comparisons (more/upward; non-directional; and less/downward | Initial questionnaire and 14-day diary to log time spent Measures: CES-D; INCOM for FB; and time spent (15-min increments up to 1 hr then 30-min increments) | Overall mean of 6.93 logins per day and time did not predict depression, overall, but social comparison and FB login frequency predict a large proportion of depressive symptoms Time spent and depressive symptoms was uniquely mediated by upward (*r* = 0.07), nondirectional (*r* = 0.07), and downward (*r* = 0.06) social comparisons |
| 62 | Tsitsika et al 2014^75^ | 10,930 | Adolescents, 14–17, 52% female; Spain, Greece, Poland, Romania, Iceland and Netherlands | Evaluate heavy SM use and internalizing behaviors (i.e., depression, anxiety) | Measures: YSR Problem Checklist; membership; online communication; time spent; internalizing problems; and social skills | 70% using SM and 40% of users spend 2+ h/day  Significant link with 2+ h/day: internalizing problems (particularly younger) and lower activities scores Heavier use by those older was positively associated with ofﬂine social skills |
| 63 | De Choudhury et al 2013A^76^ | 376 | Child-bearing age, mean 27.2 yrs; US | Use AI/ML to explore several types of FB data and Twitter posts (2,929) to detect and predict postpartum depression | Longitudinal record of events, thoughts and emotions Measures: engagement; ego-network; emotion; and linguistic style (e.g. LIWC) | Depressive signals were observable in tweets made and predict models can classify mothers who will change significantly following childbirth with an accuracy of 71%, using observations about their prenatal behavior, and as accurately as 80-83% when leveraging the initial 2-3 weeks of postnatal data |
| 64 | Feinstein et al 2013^77^ | 268 | Youth, mean 19.6 yrs; 62% female, US, Asian 42%, Caucasian 40%; US | Examined whether negative comparison in FB leads to increases in depressive symptoms, and if this is mediated by rumination | Cross-sectional (with 3-wk follow-up) Measures: SCRS; RRS; and CES-D | Negative comparison increases depression and may place lead to rumination and, in turn, more depressive symptoms Social comparison was significantly associated with increases in rumination, which, in turn, were significantly associated with depressive symptoms; depression did not mediate rumination |
| 65 | Jelenchick et al 2013^78^ | 190 | College students, mean 18.9 yrs; 58% female; 91% Caucasian; US | Assess SNS use and depression | Cross-sectional, online survey with EMA Measures: PHQ | 53% used SNSs for 30 min, 39% between 30 min-2 h and 16.8% more than 2h The mean PHQ score was 5.4 (SD 4.2); no associations were seen with SNS use and moderate to severe depression |
| 66 | Koc et al 2013^79^ | 447 | Adults mean age 21.6 yrs; technical teacher education college students; 22% female; Turkey | Explored depression, anxiety and insomnia as positive predictors of FB addiction and behavioral, demographic and psychological health predictors | Cross-sectional Measures: usage (experience, frequency, duration, and setting); motives (MFUS, 11 questions); and FAS (8 questions; Likert) | Most or 58.5% had less than 3 years of FB experience via home or dormitory (70.1%), Internet cafe (13.3%), a friend’s or relative’s home (6.2%), school (5.2%) or mobile (2.7%) Frequency: 49.6% logged in daily and 41% logged on a few times a week; mean time usage was 7 h/wk Predictors: mean FAS 13.6 (s.d. 5.92); weekly time commitment, social motives, severe depression, and anxiety and insomnia |
| 67 | Kross et al 2013^80^ | 82 | Young adults, mean yrs 19.5; 65% female, 60.5% European Am, 28.4% Asian, 6.2% AA; US | Text-messaged people five times per day for two weeks to evaluate FB use and subjective well-being, depression and anxiety | Cross-sectional with 2 wk follow-up Used experience-sampling for reliable measurement of i*n-vivo* experience | The more people used FB at one time point, the worse they felt the next check-in; the more they used FB over two weeks, the more their life satisfaction levels declined over time Non-predictors: interacting with other people, size of a FB network, perceived supportiveness, motivation for using FB, gender, loneliness, self-esteem or depression |
| 68 | Landoll et al 2013^81^ | 216 | Youth, mean 19.1 yrs; 53% female, 55% White, 25% Hispanic, 8% Black, 12% Asian; US | Evaluate peer aversive experiences | Cross-sectional Measures: CES-D; SAS-A; SN-PEQ (12 items via focus groups’ preliminary work) | The SN-PEQ – 10 items – demonstrated strong factorial invariance and a single factor structure that was distinct from other forms of peer victimization; internal consistency high for relational peer victimization and moderate for relational and overt peer victimization; validity supported |
| 60 | Landoll et al 2013^81^ | 214 | Adolescents, mean 15.7 yrs; 54% females, 75% Hispanic; 10% White; 12% Black, 3% Asian; US | Evaluate peer aversive experiences and develop/assess the SN-PEQ | Cross-sectional Measures: CES-D; SAS-A; and SN-PEQ | Supported SN-PEQ reliability and validity Negative SM experiences were associated with youths’ symptoms of social anxiety and depression, even when controlling for traditional peer victimization Cyber victimization 78%, relational peer victimization 69% and overt peer victimization 39% were common |
| 70 | Park et al 2013^82^ | 55 | Youth, mean 24.4 yrs; college and graduate students; 27% female; Korea | Use AI/ML to develop a Web application (EmotionDiary) and identify depressive symptom–related features | Advanced Institute of Science and Technology Measures: CES-D; and HAM-D | Response (i.e., number of app tips viewed and app points) was positively correlated with depression, whereas the number of friends and location tags was negatively correlated with the CES-D scale for both depressed and non-depressed |
| 71 | Wright et al 2013^83^ | 361 | Youth, mean 20.3 yrs; 54% female; 77% White, 8.6% Native Am, 4.4% Hispanic, 3.6% Asian Am; US | Examined the influence communication competence on FB and face-to-face support networks on depression and satisfaction | Used the Relational Health Communication Competence Model Measures: CES-D; Internet motives; CCS; and CMC competencies | 206 individuals single, 138 were dating, 14 married, and 2 divorced; average 2 h/wk FB and the 560 friends Interpersonal more than social integrative motives predicted in-person and CMC competence, social support satisfaction and lower depression scores |
| 72 | Dumitrache et al 2012^84^ | 76 | Adolescents, 16–18; 68% female; Australia | Evaluate SM, depression and identity on FB | Cross-sectional Measures: BDI | Significant correlations between depression, number of items about identity |
| 73 | Locatelli et al 2012^85^ | 251 | Youth, mean 18.7; 72% female, students; US | Examines the valence and frequency of FB status updates as predictors of subjective well-being: life satisfaction, physical health and depression | Online survey for 12.33 min Measures: use; CES-D; RRS; FB Questionnaire; SWLS; and CHIPS | 93% had had an account for > a year, and over half > 3 years; 62.5% spent an hour+/day and 94% used the Status Update feature 14.3% daily or 56.2% weekly Valence and frequency of status updates predict rumination which mediates subjective well-being more than FB status updates mediate impact of rumination on subjective well-being |
| 74 | Pantic et al 2012^86^ | 160 | Adolescents in high school, mean 18.0 yrs; 61% female; Serbia | Evaluate relationship between SM time spent, depression: data height and weight, gender, time spent watching TV, and sleep duration | Cross-sectional, anonymous Measures: structured questionnaire; and BDI-II | Average daily SM time spent was 1.86 h, watching TV was 2.44 h; body mass index 21.84 and sleep duration was 7.37 h Minimal 104 students, mild 46 and moderate depression in 10 Statistically significant positive correlation *r* = 0.15 was found between BDI-II score and the time spent on social networking |
| 75 | Selfhout et al 2009^87^ | 307 | Adolescents 14–17; 51% female; Netherlands | Examine use and depressive and social anxiety symptoms | Longitudinal cohort | Instant messaging T1/depression T2 *r* = −0.02 Socializing is more beneficial than Internet surfing, which is linked to low friendship quality, depression and social anxiety |
| 76 | Hwang et al 2009^88^ | 6341 | Adolescents 12–17; 51% female; Taiwan | Examine use and mood | Cross-sectional Measure: Daily Life and Depressive Mood Scale | Online communication/depressed mood *r* = 0.13 |
| 77 | van den Eijnden et al 2008^89^ | 663 | Adolescents 12–15; 52% female; Netherlands | Examine use, depression and loneliness | Longitudinal cohort over 6 months | Instant messaging was extracted, as this was much more widely used by participants (49–55%) than chatrooms (3–5%) Instant messaging correlation from T1/depression T2 *r* = 0.17 |
| 78 | Ybarra et al 2005^90^ | 1501 | Adolescents 10–17; 47% female, US | Examine use and depressive symptoms | Cross-sectional Measure: DSM-IV | 34% of chatroom and 14% Internet users had minor or major depression |

**Abbreviations:** Adolescent Stress Questionnaire (ASQ); artificial intelligence (AI); Attention Deficit Hyperactivity Disorder (ADHD); Beck Depression Inventory (BDI-II); Bergen Facebook Addiction Scale (BFAS); Bergen Social Media Addiction Scale (BSMAS); borderline personality disorder (BPD); Brief Histrionic Personality Scale (BHPS); Brief Symptoms Inventory-18 (BSI-18); negative consequences of using SNS via mobile device (CERM); Center for Epidemiological Studies Depression Scale for Children (CES-DC); character n-gram language models (CLMs); Child Depression Inventory (CDI); Cohen-Hoberman Inventory of Physical Symptoms (CHIPS); Communication Competence Scale (CCS); computer-mediated communication (CMC); Depression Anxiety and Stress Scale (DASS); Diagnostic and Statistical Manual of Mental Disorders (DSM-5); Ecological Momentary Assessment (EMA); Facebook (FB); FB Addiction Scale (FAS); FB Envy Scale (FES); FB Intensity Scale (FIS); FB Measure of Social Support (FMSS); Fear of Missing Out (FOMO); Generalized Anxiety Disorder (GAD); Global Appraisal of Individual Needs–Short Screener (GAIN-SS); Hamilton Depression Rating Scale (HAM-D or HDRS); Hospital Anxiety and Depression Scale (HADS); Information System (IS); Addiction (IA); Interpersonal Reactivity Index (IRI); Inventory of Socially Supportive Behaviors (ISSB); Iowa-Netherlands Comparison Orientation Measure (INCOM); Kiddie Schedule for Aﬀective Disorders and Schizophrenia-Present and Lifetime Version (K-SADS-PL); Latent Dirichlet allocation (LDA); Linguistic Inquiry and Word Count (LIWC); Revised NEO Personality Inventory (NEO PI- R); not applicable (NA); not specified (NS); Marlowe-Crowne Social Desirability Scale-Short Form (M-C SDS-SF); Mental Health (MH); Mental Health Inventory (MHI); machine learning (ML); Monitoring the Future (MTF) survey; Motives for FB Usage Scale (MFUS); Multidimensional Scale of Perceived Social Support (MSPSS); Narcissism Personality Inventory (NPI); Natural Language Processing (NLP) ; Need to Belong scale (NTB); Negative Self-Esteem Scale (NSES); not applicable (NA); not specified (NS); obsessive compulsive disorder (OCD); Open Trial (O); Oppositional Defiant Disorder (ODD); Patient Health Questionnaire-9 (PHQ-9); Personality Inventory for Youth (PIY); Positive and Negative Affect Schedule (PANAS); posttraumatic stress disorder (PTSD); Problematic Internet Use (PIU) Questionnaire (PIUQ-6); Problematic and Risky Internet Use Screening Scale (PRIUSS); Problematic Online Gaming (POG) Questionnaire Short-form (POGQ-SF); quality of life (QOL); Role Conflict Scale (RCS); Rosenberg Self-Esteem Questionnaire (RSEQ); Ruminative Responses Scale (RRS); Ruminative Thought Styles Questionnaire (RTSQ); Satisfaction with Life Scale (SWLS); Screen Based Media Use Scale (SBMUS); Self-Monitoring Scale (SMS); Short-Form State Anxiety Scale (SF-SAS); Single-Item Self-Esteem Scale (SISE); Social Anxiety Scale (SAS); Social Anxiety Scale for Adolescents (SAS-A); social comparison orientation (SCO); SM Use Integration Scale (SMUIS); Social Provision Scale (SPS); Spence Child Anxiety Inventory (SCIA); structural equation modeling (SEM); Short Version of the Mood and Feelings Questionnaire (SMFQ); Social Comparison Rating Scale (SCRS); social media (SM); Social Networking Intensity scale (SNI); Social Networking Peer Experiences Questionnaire (SN-PEQ); State-Trait Anxiety Inventory (STAI); Structured Clinical Interview for the DSM-IV, Patient Edition (SCID-I); Subjective Happiness Scale (SHS); World Health Organization (WHO); World Health Organization Quality of Life-Short Form (WHOQOL-BREF); Youth Risk Behavior Surveillance System (YRBSS); Youth Self Report (YSR) Problem Checklist.

Supplementary Table 2 Child, adolescent and young adult social media research study outcomes for clinical challenges like suicide, cyberbullying and sexting

| # | STUDY | N | POPULATION | OBJECTIVE/DESIGN | METHODS/MEASURES | OUTCOMES |
| --- | --- | --- | --- | --- | --- | --- |
| 1 | Khasawneh et al 2020^91^ | 150 | Adolescents, mean 15.4 yrs; global | Thematic analysis approach to code Twitter posts that explicitly referenced the blue whale challenge (along with videos and posts on those): YouTube and Twitter | Cross-sectional analysis of impact from Blue Whale (a series of 50 challenges sent directly to teens, each with increasing levels of self-harm and isolation) | Users post to raise awareness and discourage participation, express sorrow for the participants, criticize the participants, or describe an experience Depictions of suicide and self-harm in traditional media have harmful effects on vulnerable individuals, even when they describe a false or fictional behavior |
| 2 | Mori et al 2020^92^ | 239 | Adults, mean 22.4 yrs; 34.7% female; Japan | Investigate to what extent different types of SM information predict wider personality traits and attributes that relate to social behavior, decision-making, mental health, behavioral economics, empathizing–systemizing (i.e., rule), and inhibition/activation (i.e., motivation) | 24 self-reported personalities traits (and attributes) (comprising 52 subscales); mean 116 min ML models trained our different types of SM (i.e., Twitter) (network, time, word statistics, and bag of words) 3 h time intervals over 24 h Text pre-processing, affective content analysis, and bag of words model From 104 to 3200 Tweets/participant | Network data highest correlations: extraversion, social skill, communication difficulty, verbal intelligence, schizotypal interpersonal, empathic concern, happiness, detailed attention, and neuroticism ‘Favorited’ positive correlations with social aspects of autism and with verbal intelligence, but negative correlations with extraversion, empathic concern, and happiness. By contrast, ‘Reply network’ correlated positively with extraversion and empathic concern and negatively with schizotypal, attention detail and social aspects of autism Bag of word corrections were highest with verbal intelligence, socioeconomic state, imagination deficit, schizotypal, fluid intelligence, obsession, distress of delusion, social skill, alcohol use, and cigarette use. |
| 3 | Sindermann et al 2020^93^ | 494 | Adolescents, 12+ with mean 24.7 yrs; 27.5% female; from France (104), Poland (38), Spain (33), and Austria (31) | Investigate personality underpinnings of SM in broad or on a single platform, specifically, it was focused on various SM platforms | Cross-sectional FB, WhatsApp, and Instagram Measures: use disorder; IAS; and Big 5 Inventory | Impulsivity and especially extraversion were positively associated with FB, WhatsApp, and Instagram Use Disorder scores Conscientiousness (negatively) and particularly neuroticism (positively) were only significantly related to FB Use Disorder scores Non-significant associations of WhatsApp and Instagram Use Disorder scores with neuroticism |
| 4 | Corbitt-Hall et al 2019^94^ | 690 | Youth, mean yrs 20.2, 76% female; US | Investigate the odds of providing peer support in response to simulated online disclosures of suicidality Participants given the opportunity for two posts disclosing low, moderate, or severe risk for suicide | Identify predictors of support giving to friends at risk of self-harm Measures: questionnaires on depressive and anxiety symptoms, and experience with a loved one's suicidality | Only 33.6% of participants left a positive, supportive comment on one of two suicide posts. Content severity, experience with a loved one's suicide attempts; use of FB to meet people was positive Young adults vary in their propensity to provide support for a suicide disclosure online and this is driven by a combination of contextual and intrapersonal factors |
| 5 | Kircaburun et al 2019^95^ | 1143 | Adolescents (804 mean age 17.9 yrs, 29% female and 339 young adults mean age 21.5 yrs, 60% female; UK | Explore how PMSU and cyberbullying are associated with each other and to gender, age, depression, and self- esteem among high school students | Cross-sectional Measures for Study 1: CBOS; SMU; and SCS;  Study 2: same and Social Connectedness Scale; General Belongingness Scale; SDHS; and SISE | PSMU weakly correlated with depression (*r*= 0.22), gender (*r*= −0.15), age (*r*= −0.13), GB (*r*= −0.10), and self-esteem (*r*= −0.11) Depression is associated with problematic SMU and indirectly and may be associated with cyberbullying perpetration (associations weak) CBP was weakly correlated with gender (*r*= 0.20), GB (*r*= −0.18), PSMU (*r*= 0.13), depression (*r*= 0.11, *P*< 0.01), age (*r*= −0.09), and self-esteem |
| 6 | Macaulay et al 2019^96^ | 868 | Adolescents 11–13 yrs; 47.2% female; UK | Examine pupils’ bystander responses across a series of hypothetical vignettes based on traditional and cyberbullying events: (1) positive bystander responses (PBRs) will be higher in cyberbullying than traditional bullying; (2) females will exhibit more PBRs than males; and (3) PBRs vary as a function of severity of bullying | The vignettes experimentally controlled for mild, moderate and severe severity Responses: (1) seek help from a teacher, parent; (2) seek help from peer or friend; (3) direct intervention; and (4) provide comfort or emotional support | PBRs were higher in cyberbullying than traditional bullying incidents PBRs increased across mild, moderate and severe levels of bullying, for both traditional and cyberbullying, and females exhibited more PBRs for both Relative to traditional bullying, cyberbullying is characterized by anonymity and an almost unlimited capacity to reach victims Bystander response may precipitate *v*. attenuate bullying and/or its negative effects (e.g., PBRs foster help-seeking and emotional support for victims) |
| 7 | Escobar-Viera et al 2018^97^ | 11 | Middle school to college, 11–30, mean 16.7 yrs; LGBT; US | SM use (and a few other things like sexting, motivation) Linkage to depression (PHQ, CES-D, GHQ), stress and SI | 2003–17, PubMed, PsycINFO, SocINDEX | SM for coping for LGBT Cyberbullying 🡪 depression, stress and SI |
| 8 | O’Reilly Dogra and Hughes, 2018^98^ | 54 | Adolescents; 11–18 yrs; 48% female; UK | Examine potential of SM in promoting or adversely affecting well-being | Qualitative study with focus groups with 16 teachers and 8 MH providers | SM and Internet used to seek information about MH Some use SM to facilitate relaxation and reduce stress MH practitioners believe and repeat negative rhetoric of SM more than potential benefit |
| 9 | O’Reilly, Dogra, Whiteman, et al 2018^99^ | 54 | Adolescents, 11–18 yrs; UK | Examine if SM is seen as a knowledge resource and/or as a discourse to express viewpoints for well-being, its role in personal experiences, as well as use, extent and favored channels or mediums | 6 focus groups over 3 months for thematic analysis (122 s-order codes were 🡪 10 superordinate themes) | Perceived SM as a threat to mental well-being: (1) believed to cause mood and anxiety disorders for some adolescents, (2) viewed as a platform for cyberbullying, and (3) the use of SM itself was often framed as a kind of ‘addiction’ Encourage responsible behaviour on the Internet and SM |
| 10 | Pourmand et al 2018^100^ | 31 | Adolescents and young adults, mean 20.2 yrs; US | Evaluate epidemiology and risk assessment of suicide related to SM, including implications for clinicians | Pubmed/Medline, 2007–17 | Celebrity suicide could increase general rate via Twitter and Rocky Mountains has high prevalence of high-risk tweets and suicide rates Pro-suicide groups: normalization, glorification and acceptance of self-mutilation and depression Stress leads to sharing and forums and blogs can be used to share suicidal methods Clinician access to a patient’s SM may assist in identifying suicidal ideation and/or acts, but there are ethical and privacy concerns |
| 11 | Wang et al 2018^101^ | 365 | Adolescents 15.9 yrs; 61.6% female; China | Examine whether rumination and self-esteem mediated the relation between SNS addiction and depression, and if this mediating effect was moderated by self-esteem | Measures of SM addiction: FIQ for intrusion; CES-D; rumination via Chinese version of RRS; and RSES for self-esteem | SM addiction was positively associated with depression and rumination mediated this relationship The path between rumination and depression was stronger for individuals with lower self-esteem than individuals with higher self-esteem. |
| 12 | Chen et al 2017^102^ | 292 | College students, mean 19.9 yrs; 61.9% female; 77.7% White, 9.9% Asian, 7.2% Arabic, 4.5% Black; US | Test the effects of self-esteem, life satisfaction, social anxiety, privacy concerns, public self-consciousness (SC), and perceived collectivism on positive self-disclosure on SNSs | Cross-sectional | Self-esteem and perceived collectivism increased positive self-disclosure, life satisfaction Privacy concerns decreased positive self-disclosure. The effects of social anxiety and public SC were not significant |
| 13 | O’Dea et al 2017^103^ | 14 271 | Youth 22.8 yrs; Australia | Characterize the linguistic profiles of suicide-related, safe-to-ignore suicidal posts and non-suicidal-related Twitter posts | Use computer ML LIWC and regression analyses to determine differences in linguistic profiles | Suicide-related posts had higher word count, increased use of first-person pronouns and more references to death than non-suicidal-related ones Suicide-related posts had increased use of first-person pronouns, greater anger and increased focus on the present than safe-to-ignore ones |
| 14 | Salmela-Aro et al 2017^104^ | 3338 | Adolescents 12–14 yrs (1702) and 14–16 yrs; (1636); Finland | Examine: symptoms, school burnout and engagement; if use causes psychological distress and suicide attempts; and assess cyberbullying victimization as a mediator | Longitudinal with cross-sectional at two time points | Emotional engagement, school burnout and depression contribute to excessive Internet use Excessive Internet use and school burnout and school burnout leads to later excessive Internet use Burnout can decrease engagement |
| 15 | Van Rooji et al 2017^105^ | 3945 | Adolescents 12–15; 50% female; 57% pre-vocational 43% pre-college; Netherlands | Explore SM use (instant messaging, Twitter and YouTube) as a problematic ‘Internet use’ by splitting the its measurement from gaming and other Internet use | Cross-sectional Measures: CIUS; DML for mood; UCLA Loneliness Scale; NSES for esteem; and SAS for anxiety | SM and gaming were associated with depression Problematic SM use was associated with (social networking, Twitter, instant messaging and depressive mood |
| 16 | Yan et al 2017^106^ | 2625 | Adolescents, 13–18; 47% female, US | Examine screen time with SM use and unhealthy behaviors, academic performance and well-being | Cross-sectional Measures: Mental Health Scale to assess anxiety | Anxiety greater with 2+ h of use/day receiving electronic news and study materials on school days Television (2–4 h) on school days negatively affects academics; (4+ h use) reduces physical activity |
| 17 | Braithwaite et al 2016^107^ | 135 | Adolescents, 18 yrs; 63% female; 70% Caucasian, 14% Black; US | Validate the use of ML algorithms for Twitter data against empirically validated measures of suicidality: DSI-SS, INQ, and ACSS LIWC, decision tree and cross-validation analyses | Twitter feeds of Mechanical Turk (MTurk) participants were compared with validated, self-report measures of suicide risk, 2008-15 account creation | Endorsed less belongingness and more of a burden; high ratio of achievement-related words is protective ML algorithms identify the clinically significant suicidal rate in 92% of cases (sensitivity 53%, specificity 97%, positive predictive value 75%, negative predictive value 93%) |
| 18 | Cole et al 2016^108^ | 827% fema) | Adolescents 8–13 mean 10.9 yrs; 55% female; 35.8% Black, 6.0% Asian or Asian Am, 58.8% Caucasian, 9.2% Hispanic Am; US | Evaluate if cybervictimization is prospectively related to negative self- cognitions and depressive symptoms beyond other types of victimization | Longitudinal over 6 wks Measures: PVSR (victimization); RADS-2 (depression); CATS (automatic thoughts); and BYB (negative cognitive reactions to stories of victimization) | Peer victimization at either wave 1 or wave 2 (physical = 68.1%; relational = 89.8%; verbal = 87.9%; property-related = 65.8%; cyber = 63.1%). 16.1% with RADS-2 greater than 75 (mild) and 8.1% greater than 82 (moderate) depression Victimization was correlated with negative cognition and depressive symptoms; age and gender were not predictors of victimization or depression |
| 19 | Coppersmith 2016^109^ | 554 | Adults, 18–29 yrs (37%); US | To explore linguistics and emotional patterns in Twitter users with and without suicide attempt; all survivors Methods: pre-processing, *n*-gram, and emotional state | 554 users who stated that they attempted, 312 said when with exact date available for 163 users, and 125 of them had data available prior to it. | For a single point of performance for comparison, note that at roughly 10% false alarms, 70% of those who will try to take their life are identified There are differences between those who have attempted to take their life and matched controls |
| 20 | Burnap et al 2015^110^ | 1000 posts per site | Youth, mean yrs 24.1; UK | Explore suicide-related tweets using classiﬁers (lexical, structural, emotive, psychological) | Use baseline classiﬁers and built better classiﬁers using the Rotation Forest algorithm and a Maximum Probability voting classiﬁcation decision method | Classification models (10-fold cross validation after training on 90% and testing on 10%.) were used to classify tweets into relevant suicide categories F-measure of 0.728 overall (for 7 classes, including suicidal ideation) and 0.69 for the suicidal ideation |
| 21 | Muench et al 2015^111^ | 489 | Modal group 23–29 (but 18–70); 65.6% female; 84.1% White, 5% Asian, 5% Hispanic; 86.3% some college; US | Explore FB time spent, checking and addiction (neglect, social life interference and failed attempts to reduce) Outcomes FOMO, social life interactions and perception that FB users have better lives | Cross-sectional 10–15 min survey Used Amazon.com Mechanical Turk to recruit General questions and one measure *(FNSE*) | 80.8% on FB 1+ time/day and 20% 2+ h/day; mean 31–45 min/day Associations: time with checking (beta 0.46) and addiction (beta 0.28); checking with addiction (beta 0.21); addiction with FOMO (beta 0.21) and FB with better lives (beta 0.11) |
| 22 | O’Dea et al 2015^112^ | 2000 | Youth, 24 yrs; Australia | To detect the level of concern for each suicide-related tweet | Compare human coders *v*. computer ML classifier to estimate the level of concern from suicide-related tweets | Of suicide-related tweets, 14% deemed ‘strongly concerning’ and warrant further investigation Overall agreement rate was 76% (K = 0.55) but ‘strongly concerning’ tweets could not be validated |
| 23 | Sampasa-Kanyinga and Hamilton 2015^113^ | 5126 | Youth, 11–20 yrs, mean 15.2 yrs, 48% female; Canada | Examined the link between the use of SNS and psychological distress, suicidal ideation and suicide attempts, and tested the mediating role of cyberbullying victimization on these associations in adolescents | 2013 Ontario Student Drug Use and Health Survey with adjustments for age, sex, ethnicity, subjective socioeconomic status, and parental education | SNSs was associated with psychological distress (adjusted odds ratio, 95% confidence interval = 2.03, 1.22–3.37), suicidal ideation (3.44, 1.54-7.66) and attempts (5.10, 1.45–17.88); cyberbullying victimization mediated psychological distress and attempts, but only partially mediated suicidal ideation |
| 24 | Sampasa-Kanyinga and Hamilton 2015^113^ | 5329 | Middle and high school students, mean 13.1 yrs; Canada | Examines the association between SM use and cyberbullying victimization | 2013 Ontario Student Drug Use and Health Survey | 19% cyberbullied in the past 12 months: female, younger, of lower socioeconomic status, and who used alcohol or tobacco were at greater odds Increased risk of cyberbullying victimization in a dose–response manner (*P*-trend < 0.001) |
| 25 | Sampasa-Kanyinga and Lewis 2015^114^ | 753 | Adolescents, mean 14.1 yrs; 55% female; Canada | Evaluate heavy SM use and psychological distress | 2013 Ontario Student Drug Use and Health Survey Measures: Kessler K-10 Scale | Overall, 25.2% of students reported use more than 2 h every day, 54.3% reported use 2 h or less every day 2+ h/day associated with poor self-rating of MH, high levels of psychological distress and suicidal ideation |
| 26 | Zhang et al 2015^115^ | 1041 | Adults mean age 24 yrs; 62% female; China | To compare trained and inferred topics NLP algorithms via posts to predict probability of suicide using Suicide Probability Scale | Sina Weibo mblog Up to 2000 mblogs/user Chinese version LIWC model and LDA (topic model) for ML processing | Suicide hotlines and face-to-face diagnosis are effective methods, but rely on initiative by others LDA predicted suicide probability better than LIWC Inferred topics have equal, even higher, predictive power than trained topics and are more reusable |
| 27 | La Sala et al 2014^116^ | 374 | College students (190) and others (184) mean 26.2 yrs; 71.8% female; US | Examine the influence of Big Five personality traits on FB usage and examined the interactions of traits (Torgersen, 1995) | Cross-sectional Measures: use; RSES; API; NPI; UCLA Loneliness Scale; BPS; and RCBS | 79% reported using FB everyday, primarily with friends and secondarily with folks from the past Narcissism predicts time spent for both students and non-students, and number of daily logins for non-students (though superseded by agreeableness) Extraversion and Neuroticism were the strongest predictors of number of friends for students; Extraversion and Conscientiousness for non-students |
| 28 | Rodriguez Puentes and Parra, 2014^117^ | 96 | Adolescents 11–15 yrs; 52.2% female; Bogata, Columbia | Explore the relationship between the time spent and the presence of internalizing and externalizing (e.g. (aggression, rule breaking) behavior | Experimental or quasi- experimental study | Greater time spent on social networks was associated with externalizing disorders such as aggressive conduct, rule breaking and attention deficits. There was no association with depression |
| 29 | Tseng and Yang, 2014^118^ | 391 | Adolescents, 12–18; Taiwan | Investigate Internet use, web communication and social support in those with self-injurious thoughts and behaviors (SITBs) | Cross-sectional (2-phase sampling design) | Girls are more likely to have SITBs, except for suicide gestures. Internet is a risk factor for SITBs in boys but not in girls. Support from SNS can have both positive and negative effects, with different effects by gender |
| 30 | Jashinsky et al 2013^119^ | 1 208 809 users | Adolescents, youth and adults, mean 24.1 yrs; US | To compare ML with aggregate state data on suicide risk and rates | Cross-section application-programming interface, filtering by terms and geolocation | Twitter assessment of suicide risk compared favorably with rates of suicide from Vital Statistics Data Via the Centers for Disease Control and Prevention (i.e., actual suicidal rates by state) and other databases |
| 31 | Romer et al 2013^120^ | 719 | Adolescents and young adults; 14–22; 51% female; US | Explore the impact of older and newer SM use on academic, social, and MH outcomes in adolescents and young adults | Cross-sectional | Greater Internet and SM use were associated with recent depression. Information users had higher grades, participated in clubs more often, and were lowest in depression |
| 32 | Dunlop et al 2011^121^ | 719 | Adolescents and young adults 14–24 yrs; 51% female; 70% White; 13% Hispanic, 10% Black; US | Examine exposure spread about suicide and SI: (a) friend or relative; (b) newspaper; (c) Internet news site like CNN or Yahoo News; (d) video website like YouTube; (e) online forum, discussion board, or self-help website; (f) site like FB; or (g) other | Longitudinal and part of the National Annenberg Survey of Youth by the Annenberg Public Policy Center at the U. of Pennsylvania | 11% of respondents had seriously considered committing suicide in the last 12 months Sources news 64%, friends or relatives 55% and Internet news sites 44% news sites (SNS 25%, online discussion forums 15%, and video websites 15% Online discussion boards, but not SNS associated with increased SI |
| 33 | Szwedo et al 2011^122^ | 18 | Adolescents from 13.2 yrs to 20.3 yrs; 56% Caucasian, 33% Black and 11% other; family income at T1 $40 000–59 999; US | Determine if negative interactions with mothers at age 13 leads to more negative peer interactions at 20 (blaming others for disagreement, pressuring others to change positions, ignoring or interrupting the others, hostile or critical statements) and greater likelihood of preferring online friendships | Cross-sectional with 7 year f/u 89 had SM FB; 63 granted access permission) Measures T0: CDI; PEI (9 items, social behavior) Measures T1: BDI; and SAS-A Standardized system to code the interactions for behaviors (i.e., adolescent-family interaction) | Poor adolescent relationships with the mother predicted preference for online communication, likelihood of forming friendships with people met online, and poorer quality of online relationships at age 20 Females receive more supportive messages Depressive symptoms at age 13 predicted a greater preference for online communication, but depressive symptoms at age 20 reduced preference for online communication and social withdrawal |
| 34 | Mitrofan et al 2009^123^ | 86 | Children (7–11); UK | Evaluate if aggression seen on SM or Internet (yell, verbal threat, hit others, slam door, fight with others) and media is linked to aggressive behavior | Children outpatients (47) and parent(s) (39) using qualitative semi-structured interview; multiple aggression scales | Seeing aggression influences others but not themselves Carers regard aggression as resulting from a combination of inner and environmental factors and see aggression in real-life as having more impact than television/video games |

**Abbreviations:** Acquired Capability for Suicide Scale (ACSS); Application-programming interface (API); Beck Depression Inventory (BDI-II); Behind Your Back protocol (BYB); borderline personality disorder (BPD); Boredom Proneness Scale (BPS); Center for Epidemiological Studies Depression Scale for Children (CES-DC); character n-gram language models (CLMs); Child Depression Inventory (CDI); Children’s Automatic Thoughts Scale (CATS); Cognitive Triad Inventory (CTI); Compulsive Internet Use Scale (CIUS); computer-mediated communication (CMC); Cyberbullying Offending Scale (CBOS); Depressive Mood List (DML); Depressive Symptom Inventory–Suicide Subscale (DSI-SS); Ecological Momentary Assessment (EMA); FaceBook (FB); FB Intrusion Questionnaire (FIQ); FB Measure of Social Support (FMSS); Fear of Missing Out (FOMO); Generalized Anxiety Disorder (GAD); General Health Questionnaire (GHQ-28); Hamilton Depression Rating Scale (HAM-D or HDRS); Hospital Anxiety and Depression Scale (HADS); Information System (IS); International Personality Item Pool (IPIP); Internet Addiction (IA); Interpersonal Needs Questionnaire (INQ); Interpersonal Reactivity Index (IRI); Inventory of Socially Supportive Behaviors (ISSB); Latent Dirichlet allocation (LDA); Leisure Time Exercise Questionnaire (LTEQ); Lesbian Gay Bisexual Trans (LGBT); Linguistic Inquiry and Word Count (LIWC); not applicable (NA); not specified (NS); machine learning (ML); Mental Health (MH); Mental Health Inventory (MHI); Narcissism Personality Inventory (NPI); Natural Language Processing (NLP); Negative Self-Esteem Scale (NSES); not applicable (NA); not specified (NS); obsessive compulsive disorder (OCD); Open Trial (O); Patient Health Questionnaire-9 (PHQ-9); Problematic Social Media Use (PMSU); Pupil Evaluation Inventory (PEI); quality of life (QOL); randomized controlled trial (RCT); Revised Cheek and Buss Shyness Scale (RCBS); Reynolds Adolescent Depression Scale – Version 2 (RADS-2); Role Conflict Scale (RCS); Rosenberg Self-Esteem Questionnaire (RSEQ); Ruminative Responses Scale (RRS); Short Depression-Happiness Scale (SDHS); Single-Item Self-Esteem Scale (SISE); Social Anxiety Scale (SAS); Social Anxiety Scale for Adolescents (SAS-A); social media (SM); Social Networking Intensity scale (SNI).

Supplementary Table 3 Child, adolescent and young adult social media research study outcomes for health behavior and well-being

| # | STUDY | N | POPULATION | OBJECTIVE/DESIGN | METHODS/MEASURES | OUTCOMES |
| --- | --- | --- | --- | --- | --- | --- |
| 1 | Twenge et al 2020^12^ | 221 096 | Adolescents, 13–18 yrs; Open Science in UK; and the YRBSS and Monitoring the Future (MTF) in US | Examine gender differences in associations between digital media use (device, smartphone, computer), speciﬁc digital media activities like SM, texting, gaming, other), and psychological well-being | Cross-sectional Measures: WEMWBS in UK; depression and SI in YRBSS; and happiness in MTF | Girls spent more time on smartphones, SM, texting, computer; boys spent more time gaming and devices Light users (0.5 h) of digital media were slightly higher in well-being than non-users Heavy users (>5 h) were often twice as likely as low users (1–2 h) to have low well-being, mental health issues and suicide risk factors; larger effects for girls than for boys |
| 2 | Alcott et al 2019^124^ | 2897 | Young adults <30, mean 24.2 yrs; US | Compare outcomes for substitute time use, social interaction, online time, well-being and loneliness | Comparison groups: one deactivate FB 4 weeks *v*. usual longitudinal use Measures: SHS; and SWLS | Experimental group showed small increases in well-being measured retrospectively; no changes in well-being or loneliness reports |
| 3 | Orben and Przybylski, 2019A^125^ | 17 247 | Adolescents, mean 15.5 yrs; UK, US and Ireland | Evaluate screen time and psychological well-being using software composition analysis | Measures: SDQ by teens and parents; SMFQ; RSES; CDI; and questions about digital engagement | Analytical pathways suggest small negative association measured throughout the day or particularly before bedtime, *r* = 0.1 |
| 4 | Orben and Przybylski, 2019B^126^ | 355 358 | Adolescents, 10-15; Open Science in UK; and the YRBSS and MTF in US | Evaluate digital technology use and effects on life satisfaction and well-being | Compare 3 large-scale exemplar datasets | The association between digital technology use and adolescent well-being is negative, but small, explaining at most 0.4% of the variation in well-being |
| 5 | Pope et al 2019^127^ | 38 | College students, mean 21.5 yrs; US | Twice/week FB-delivered health education and accelerometer-estimated physical activity, dietary behavior and psychosocial/ physiological outcomes | Comparison of experimental (social cognitive theory- and self-determination theory) to control group | Intervention adherence was 86%, with retention of 92.1% Participants had 1–3X/week, with 4.2- and 1.6-min/day increases in moderate-to-vigorous exercise, respectively, at six weeks—partially maintained at 12 weeks; similarly decreased body weight (−0.6 kg to −0.5 kg) and increased self-efficacy, social support, and intrinsic motivation |
| 6 | Rasmussen et al 2019^128^ | 546 | College students, mean age 20.8 yrs; 66% female; 73% Caucasian, 13% Hispanic, 7% Black, 2% Asian, 1% Am Indian/Alaska Native; US | Explore the serially mediated relationship between emerging adults’ SM use and mental well-being | Cross-sectional Measures: -DERS -Perceived social stress scale -Mental health issues: 14 questions related to their state of mind | SM use is not associated with MH problems, nor is emotional regulation Emotional regulation is associated with perceived stress Perceived stress is associated with MH problems SM use does not indirectly predict MH problems as mediated by perceived stress or emotional regulation SM use may be a risk factor for MH struggles among emerging adults and that SM use may be an activity which emerging adults resort to when dealing with difficult emotions |
| 7 | Coyne et al 2018^129^ | 681 (457) | Families of subjects age 11–14; 53% female; US | Evaluate SM use over time and predictors of depression and physical aggression | Cross-sectional 2 wks | Moderate SM users had higher levels of self-regulation and lower levels of overall media use *v*. the other 2 classes (peak users and increasers), which had higher levels of depression and physical aggression |
| 8 | Hunt et al 2018^130^ | 143 | College, mean age 20.1 yrs; US | Compare outcomes for FOMO | Comparison groups: 10 min/day *v*. usual use | Both groups showed decreases in anxiety and FOMO; only the experimental group showed additional decreases in loneliness and depression |
| 9 | Vanman et al 2018^131^ | 138 | Youth (18–30), mean 22.4 yrs; 63% female; US Baseline friends 506.11, 7.62 logins/day and 2.85 mean hrs use | Examine pre- and post-evaluation of perceived stress and well-being, and salivary cortisol (2–5 pm) when stop FB 5 days | Compare two groups: give up FB for 5 days or continue to use Measures: SWLS; PSS; PANAS; and SELSA-S | More friends associated with more daily hrs, active use, greater positive affect, and less SELSA-S family loneliness Active use associated with higher SWLS, lower SELSA-S social loneliness, and lower SELSA-S family loneliness FB break lowered cortisol levels, life satisfaction, perceived stress and lower SWLS well-being |
| 10 | Wright et al 2018^132^ | 211 | Adults, 18–29 yrs; 675 female, university and working; Australia Baseline friends 487, used FB for 6 yrs and 14 h/wk | Use AI/ML to develop a preliminary inventory of FB false self-presentation behaviors | Cross-sectional Measures: frequency of engagement; false self-presentation; self-esteem; social influences; well-being; depression; anxiety; and stress | Presence of two distinct false self-presentation behaviors: lying (e.g., untruthful status updates, profile creation) and liking behaviors (e.g., liking posts dishonestly), each associated with different predictors and outcomes. Moral norms significantly predicted lying behaviors; and age, self-esteem, group norms, and moral norms significantly predicted liking behaviors |
| 11 | Frith et al 2017^133^ | 39 | College students, mean 22.7 yrs; 68% female; US | Evaluate an 8-week dynamic *v*. static FB SM intervention on physical activity and anxiety behaviors | RCT comparing dynamic (active; 1–2 items daily) *v*. static (passive; 96 items at the beginning) | Anxiety was reduced for the dynamic, but not the static group, along with a significant group-by-time interaction and increase in physical activity A statistically significant inverse correlation between mean total physical activity change and mean change in anxiety |
| 12 | Sun et al 2017^134^ | 196 | Chinese college students, mean 19.9 yrs; Hong Kong | Compare a peer-led, SM-delivered, safer sex intervention with a sexual health website and weekly emails to remind them to visit the website | RCT of peer-input education materials and a peer-led, safer sex FB group, 6 weeks within Hong Kong Family Planning Association, 2004 | Intervention group: more satisfying online experiences; higher level of online-visiting frequency; attitudinal improvement in condom use; and behavioral skills Increased online-visiting frequency was associated with better contraceptive use behavioral intention, better behavioral skills, and more frequent condom use |
| 11 | Ehrenreich et al 2016^135^ | 125 | Adolescents all 18 yrs; 44.8% female; 2.4% Asian, 20.8% Black, 16.8% Hispanic, 52.8% White; US | Examine internalizing symptoms (i.e., depression, anxiety, loneliness) related to FB communications and responses received | Longitudinal over 2 months Measures: CBCL-YSR; PANAS; eliciting or offering support; somatic complaints; and peer comment frequency | Mean posts 60.2 overall and for girls 88 *v*. boys 37 For girls, internalizing symptoms predicted: negative affect, somatic complaints and eliciting support; they also predicted receiving more peer comments expressing negative affect, and peer responses offering support |
| 13 | Marder et al 2016^136^ | 379 | Young adults, mean age 22 yrs; 69% female; UK | Evaluate if online presentation is constrained to audience standards strictest (i.e., lowest common denominator effect (LCDE) *v*. strongest audience effect (SAE; standards and value combined) | Self-presentation Measures: strictest audience standards (SAQ); SAE, self-censoring and self-cleansing Groups: close friends, relational partners, employer, guardians, acquaintances | There was a signiﬁcant overall difference across audience groups on perception of strictness and strength; SAE predicts behavioral constraint and also social anxiety better than LCDE Employers are perceived as the strongest audience as well as joint strictest with parents/guardians For self-censorship and self-cleansing, the SAE was positively associated with greater caution when communicating content; the LCDE did not signiﬁcantly predict well |
| 14 | Gunnell et al 2015^137^ | 1160 | Adolescents, mean 13.5 yrs; 60.5% female; 74.1% Caucasian; 54.9% both parents college; Canada | Examine the bidirectional relationship between physical activity (PA), screen time (ST), and symptoms of anxiety and depression; control for sex, ethnicity, BMI, birth year, and parents’ education | Longitudinal: 4 assessments over 11 yrs Measures: LTEQ for PA; questionnaire for ST: none; <1 h; 1–3 h; 3–5 h; 5–8 h; >8 hrs; CDI; and MASC-10 | PA decreased over time whereas ST and symptoms of depression and anxiety increased over time Higher anxiety was associated with initially higher ST and initially lower PA independent of initial symptoms of depression; associated with increases in ST and increases in depression Higher depression was associated with higher initial ST and predicted decreased PA (b = −0.28) |
| 15 | Lin et al 2015^138^ | 890, 1109 | Youth, mean 22.6 yrs; 77.5% college; 58.3% female; Taiwan  Youth, mean 35.7 yrs; average 713.5 min/wk | Explore FB use, attachment orientations, and online and offline social capital Survey, connectedness statements, Likert scales, | Compare University student (study 1) and national samples (study 2) | Secure: positively associated with online bonding, bridging and all capital and offline bridging capital  Avoidant: negatively associated with online bonding capital Anxious–ambivalent: had a direct association with online bonding capital and an indirect effect on all FB capital |
| 16 | Neubaum et al 2015^139^ | 60 | College, 18–30, mean 23.8 yrs; 50% female; Germany | Explore social closeness based on social networking activity levels: consumption, participation and production (i.e., active generation of content) | Quasi-experimental: spent ten minutes on FB and then reported experiences Measures: need to belong; perceived social closeness; and emotional states | Mean 7.82 min consuming content and 3.13 min on participation; only two performed productive activities Time spent interacting with other users (e.g., commenting on updates), led to feeling closer and predicted positive emotional states after use Needing to belong may predict participation |
| 17 | Rae et al 2015^140^ | 119 | College students 75.4% 18–21 and 73.6% female; 85.2% white, 7.4% Hispanic, and 7.4% multiracial; US | Investigated whether these contradictory findings of quantity of FB use could be explained by considering users’ motivations for access | Cross-sectional Measures: Motivation 11 questions; and MHI for well-being | The relationship of quantity of FB use on psychological well-being was moderated by the motivation of the user Quantity of use was associated with higher levels of psychological well-being among users that accessed it for friendship purposes but was negatively associated with psychological well-being among users that accessed it for connection purposes (e.g., making new friends) |
| 19 | Verduyn et al 2015^141^ | 84 | Youth, college students, mean 19.9 yrs; 62% female; US | Examine if FB usage predicts declines in subjective well-being over time by cueing people to use it in lab | Compare active *v*. passive groups | Motivation for using FB: keep in touch with friends (77% yes); to find new friends (19%); to share good things with friends (75%); to share bad things with friends (34%); to obtain new information (76%); or other (14%) Passive use participants displayed a significant drop in affective well-being– in 9% of participants – at the end of the day relative to both their baseline and post-manipulation affect levels; active use participants did not |
| 20 | Verduyn et al 2015^141^ | 89 | Youth college students, mean 20.2 yrs; 69% female; 53% European Am, 34% Asian 8% Black, and 5% other; US | Examine if FB usage predicts declines in subjective well-being over time in the field Text-messaged 5X/d at random times between 10 am-midnight for 6 consecutive days | Compare active *v*. passive groups Measures: SWLS; Revised UCLA Loneliness Scale; and SPS | Responded to 80% of the text messages People interacted ‘directly’ with other people more frequently than any other type of social interaction, but used used FB passively more than they used it actively or non-FB SM Motivation for using FB: keep in touch with friends (95% yes); find new friends (13%); share good things with friends (52%); share bad things with friends (7%); obtain new information (68%); or other (20%) Passive FB usage undermines affective well-being in 5% |
| 21 | Blomfield-Neira and Barber, 2014^142^ | 1819 | Adolescents 13–17 mean 14.6 yrs; 55% female; 83.4% Caucasian, 7.2% Asian, 1.5% Black and 1.3% Aboriginal and Torres Strait Islander; 6.6% Other; Australia | Investigate whether there was a relationship between adolescents’ use (none to several times/day) of SNSs and their social self-concept, self-esteem, and depressed mood | Cross-sectional in Youth Activity Participation Study (adolescents’ experiences during their leisure time) Measures: use; investment; indicators of adjustment; self-esteem; and mood | Females and males: 76% *v*. 67% have profile, 27% *v*. 46% public and 18% *v*. 33% have untrue information, respectively. Females use SM more than in-person, flirt less, are more invested, and have lower sense of self-concept than males No link between SM frequency and depressed mood, but SM investment did predict depressed mood |
| 22 | Coyne et al 2014^143^ | 491 | Families of adolescents, 12–17, mean 14.4 yrs; 53% female; 72% Caucasian, 8% were Black, and 20% were multi-ethnic; average annual income was $60,000 per year; US | Examine association between parent–child use of SM and feelings of connection | Cross-sectional Measures: SM use (0 to 8+ h); time watching TV, Internet, or listening to music; parental connection PSDQ; internalizing and delinquency; relational aggression; and prosocial behavior (VAIS) | Females reported higher overall levels of SM than did males; social networking between child and parent does occur but on a relatively rare basis (e.g., 50% of reported only occasional use; 19% multiple times per month and 16% reported using such sites with their parents every day Social networking with parents was associated with increased connection and this mediated the relationship between social networking with parents and depression; use without parents was associated with depression |
| 23 | Farquhar et al 2014^144^ | 336 | College students from 5 universities, mean 19.9 yrs; 70% female; 80% White; 66%; US | Examined how religiosity, network homophily, and self-monitoring relate to social and FB-specific anxiety, role conflict, and FB Intensity | Cross-sectional correlational analysis Measures: FIS; SAS; RCS; and SMS | Link between FB use and anxiety, and religiosity and anxiety  Role Conflict correlates with FB Intensity, FB-specific Anxiety, and Social Anxiety Those who prefer a literal interpretation of the Bible attend church more frequently, pray more and have higher anxiety Higher self-monitoring participants have a less homophilous FB network and are less likely to share their religious views |
| 24 | Vogel et al 2014^145^ | 145 and 128 | College, mean 19.6 yrs (study 1) and 19.1 yrs (study 2); 73% and 68% female; US | Examine in Study 1 if SM use is associated with lower self-esteem and in Study 2 the impact of temporary exposure to SM profiles on state self-esteem and self-evaluation | Clinical trial with study 1: correlational approach for Study 1 and an experimental approach for Study 2 | Study 1: FB users had poorer trait self-esteem which was mediated by upward comparisons Study 2: state self-esteem and relative self-evaluations were lower when the target person’s profile contained upward comparison information (e.g., a high activity social network, healthy habits) than when the target person’s profile contained downward comparison information |
| 25 | Tsugawa et al 2013^146^ | 50 | Young adults, 21-27, mean 24.1; 50% female; Japan | Explore if an analytic (i.e., multiple regression) model is effective in estimating depressive tendencies from users’ activities on a SN | Cross-sectional Examine if user’s activities in Twitter estimate a depressive tendency 3200 tweets/person | Medium positive correlation (*r* = 0.45) between the Zung Self-Rating Depression Scale and the model estimations, when candidate words ≤20 99 words correlate with Zung scores: even if, very, workplace, hopeless, disappear, too much, sickness, bad and hospital |
| 26 | Burke et al 2011^147^ | 415 | College students and adults, mean 33.7 yrs; 63% female Sample more: time spent 1.7 h/d *v*. 0.5, friends 185.6 *v*. 170.0, women; US | Evaluate: (1) the impact of: SM activity on social capital and loneliness; and (2) individual differences among users, including social communication skill and self-esteem | Longitudinal over 9 months Capital survey items: 5 bonding, 10 bridging, social communication 10, and self-esteem 7 | Active SM use increases social capital and decreases loneliness; passive doesn’t Person-to-person exchanges – not passive consumption or broadcasting - increases in bridging social capital Receiving messages from friends is associated with increases in bridging social capital; those with lower social fluency draw value from passively consuming news |
| 27 | Burke et al 2010^148^ | 1193 | College students and adults, mean 33.7 yrs; 63% female; international | Validate SM self-report scale; study in an older, international populations; and find activities associated with social capital and loneliness | Cross-sectional Measures: FIS; and Hadoop distributed computation platform | Directed communication is associated with greater feelings of bonding social capital and lower loneliness, but has a modest relationship with bridging social capital (overall friend network size) Consumers of greater levels of content report reduced bridging and bonding social capital, and increased loneliness |
| 28 | Ellison et al 2007^149^ | 286 | College students, mean 20.1 yrs; 66% female; 87% White; US | Examine the relationship between use of FB and formation/maintenance of social capital Meet new people online *v*. existing offline contacts | Cross-sectional study Measures: FIS; RSES; SWLS; and social capital | A strong association between use of FB and the three types of social capital, with the strongest relationship being to bridging social capital Less intense FB users, students with low satisfaction of college life also reported having much lower bridging social capital than those who used FB more intensely |

**Abbreviations:** artificial intelligence (AI); Center for Epidemiological Studies Depression Scale for Children (CES-DC); character n-gram language models (CLMs); Child Behavior Checklist-Youth Self Report (CBCL-YSR); Child Depression Inventory (CDI); Cognitive Triad Inventory (CTI); Facebook (FB); FB Addiction Scale (FAS); FB Envy Scale (FES); FB Intensity Scale (FIS); Fear of Missing Out (FOMO); Information System (IS); Internet Addiction (IA); Leisure Time Exercise Questionnaire (LTEQ); machine learning (ML); Mental Health (MH); Mental Health Inventory (MHI); Motives for FB Usage Scale (MFUS); Multidimensional Anxiety Scale for Children-10 (MASC-10); not applicable (NA); not specified (NS); Open Trial (O); Parenting Styles and Dimensions Questionnaire-Short Version (PSDQ); Positive and Negative Affect Schedule (PANAS); quality of life (QOL); randomized controlled trial (RCT); Role Conflict Scale (RCS); Satisfaction with Life Scale (SWLS); Self-Attributes Questionnaire (SAQ); Self-Monitoring Scale (SMS); Social Desirability Scale (SDS); Social Provision Scale (SPS); Short Version of the Mood and Feelings Questionnaire (SMFQ); Social Comparison Rating Scale (SCRS); Social and Emotional Loneliness Scale for Adults (SELSA-S); social media (SM); social network site (SNS); Strengths and Difficulties Questionnaire (SDQ); Subjective Happiness Scale (SHS); Values in Action Inventory of Strengths (VAIS); Warwick-Edinburgh Mental Well-Being Scale (WEMWBS); Youth Risk Behavior Surveillance System (YRBSS).
